# Dopant triggered atomic configuration activates water splitting to hydrogen

**DOI:** 10.1038/s41467-023-37641-3

**Published:** 2023-04-21

**Authors:** Rui Wu, Jie Xu, Chuan-Lin Zhao, Xiao-Zhi Su, Xiao-Long Zhang, Ya-Rong Zheng, Feng-Yi Yang, Xu-Sheng Zheng, Jun-Fa Zhu, Jun Luo, Wei-Xue Li, Min-Rui Gao, Shu-Hong Yu

**Affiliations:** 1 Division of Nanomaterials & Chemistry, Hefei National Research Center for Physical Sciences at the Microscale, New Cornerstone Science Laboratory, Department of Chemistry, Institute of Biomimetic Materials & Chemistry, Anhui Engineering Laboratory of Biomimetic Materials, University of Science and Technology of China, Hefei, 230026 Anhui P. R. China; 2 Institute of Functional Nano and Soft Materials (FUNSOM), Jiangsu Key Laboratory for Carbon-Based Functional Materials & Devices, Soochow University, 215123 Suzhou, P. R. China; 3 Department of Chemical Physics, School of Chemistry and Materials Science, University of Science and Technology of China, Hefei, 230026 Anhui P. R. China; 4 Shanghai Synchrotron Radiation Facility, Shanghai Advanced Research Institute, CAS, 201210 Shanghai, P. R. China; 5 National Synchrotron Radiation Laboratory, University of Science and Technology of China, 230029 Hefei, P. R. China; 6 School of Materials Science and Engineering, Tianjin University of Technology, 300384 Tianjin, P. R. China

**Keywords:** Electrocatalysis, Electrocatalysis

## Abstract

Finding highly efficient hydrogen evolution reaction (HER) catalysts is pertinent to the ultimate goal of transformation into a net-zero carbon emission society. The design principles for such HER catalysts lie in the well-known structure-property relationship, which guides the synthesis procedure that creates catalyst with target properties such as catalytic activity. Here we report a general strategy to synthesize 10 kinds of single-atom-doped CoSe_2_-DETA (DETA = diethylenetriamine) nanobelts. By systematically analyzing these products, we demonstrate a volcano-shape correlation between HER activity and Co atomic configuration (ratio of Co-N bonds to Co-Se bonds). Specifically, Pb-CoSe_2_-DETA catalyst reaches current density of 10 mA cm^−2^ at 74 mV in acidic electrolyte (0.5 M H_2_SO_4_, pH ~0.35). This striking catalytic performance can be attributed to its optimized Co atomic configuration induced by single-atom doping.

## Abstract (web summary)

Designing high performing low-cost nanomaterials for catalyzing hydrogen evolution reaction (HER) remains challenging. Here, the authors report a volcano-shape correlation between HER activity and cobalt atomic configuration in single-atom doped CoSe2-DETA (DETA = diethylenetriamine) nanobelts toward achieving high performance.

## Introduction

Hydrogen is considered as a clean and renewable alternative to replace traditional fossil fuels^1^. Electrocatalytic water splitting for hydrogen production via hydrogen evolution reaction (HER) is an ideal strategy to meet the criteria of future sustainable society^2^. To date, the benchmark HER electrocatalyst is still Pt and Pt-based materials, whereas their high price and scarcity hinder the large-scale application^3^. Thus, the rational design of cost-effective and highly efficient HER electrocatalysts is emergent. Recent advances^4^ in transition metal chalcogenides^5–11^, phosphides^12–17^, nitrides^18,19^, borides^20–23^, carbides^21,24,25^ and oxides^26^ have demonstrated atomic modulation of electronic structure to optimize HER activity^27,28^. Among them, ion doping represents a powerful strategy to boost HER performance^10,29,30^. Universally, doping with foreign atoms will change the local coordination and modulate the electronic structure, which can result in enhanced catalytic performance^11^. Nevertheless, insights, especially with assist of advanced techniques, are still urgent to elucidate the activity enhancement mechanism in depth.

Currently, single atom catalysts (SACs) with isolated metal atoms dispersed on substrate have emerged as ideal heterogeneous catalysts^25,31–36^. Due to the low coordination environment of active metal center and strong metal-support interaction, SACs show excellent performance in fields of oxidation reactions, water-gas shift and hydrogenation reactions^37^. Very recently, with advantages of SACs and doping, single-atom doped catalysts (SADCs), consisting of dopants which atomically substitute original atoms, have attracted intensive interests^29,38,39^. The catalytic performance of SADCs can be adjusted in two ways: single atom dopants as new active sites or modifying electronic structure of former active sites. In spite of several examples of SADCs for electrocatalytic hydrogen generation, their HER activity is still far from the commercial Pt/C catalysts. Currently, the development of highly active and stable SADCs-based HER catalysts remains challenging.

Previous reports have verified cobalt diselenides (CoSe_2_)-where Co atoms are octahedrally bonded to nearby Se atoms with corner-shared structure-as cost-effective and efficient HER catalysts^28,40–43^. Especially, our group has reported several strategies to prepare CoSe_2_-based electrocatalysts with HER activity comparable to Pt/C, including material grafting, anion-induced phase transition and phase mixing^42^. However, to date, few reports have focused on CoSe_2_-based SADCs. Such materials with general synthetic methodology as well as superior HER performance far remain unexplored.

Herein, we describe a facile single atom doping strategy to modify HER activity of low-cost CoSe_2_-DETA (DETA = diethylenetriamine) nanobelts by substituting Co with 10 kinds of other metal ions (donated as M-CoSe_2_-DETA, M = Cr, Mn, Fe, Ni, Zn, Mo, Cd, W, Bi, and Pb). It’s reported that ligands like amine molecules favor shape-controlled synthesis^44,45^ and catalytic promotion through surface coordination adjustment^46,47^. Herein, DETA, an amine molecule, can serve as a template to prepare nanobelt-like products^44^. Compared with the only existence of Co-Se bonds in pure CoSe_2_^40^, once templated by DETA, Co-Se bonds and Co-N bonds co-exist in CoSe_2_-DETA. When doped with single-atom ions, the dopants can change the coordination number of Co-Se bonds and Co-N bonds, thus modulating the electronic structure of Co atoms as well as HER activity of products. We summarized a volcano relationship between HER catalytic performance and coordination environment of Co atoms (ratio of Co-N bonds to Co-Se bonds), while Pb-CoSe_2_-DETA shows the best HER behavior which is even close to the state-of-the-art commercial Pt/C catalysts owning to its optimized Co local coordination.

## Results

### DFT study of thermodynamic stability

**Fig. 1:**
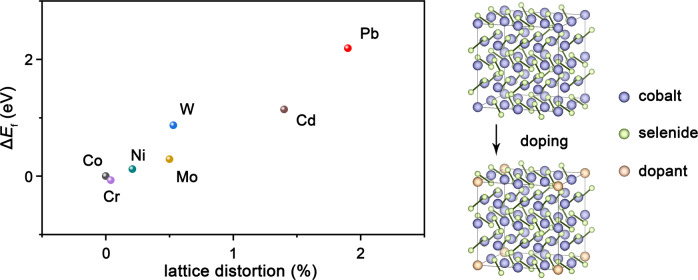
DFT thermodynamic stability study. Calculated formation energies of various cation doped CoSe_2_ as a function of lattice distortion, dopant concentration: 3.125 at%. (Right) Structures of pure pyrite CoSe_2_ and cation doped CoSe_2_. The balls in purple, green and orange represent Co, Se, and dopants, respectively.

We first performed DFT calculations to establish the thermodynamic stability of CoSe_2_ lattices with Co atoms substituted by different cations. We started from pure pyrite CoSe_2_ structure. Using CoSe_2_ as reference, we then calculated the formation energy and corresponding lattice distortion of M-CoSe_2_. By replacing Co atom with several typical cations, a correlation between formation energy and lattice distortion was found, where a more severe distortion generally corresponds to a more positive formation energy (Fig.1). For Pb-CoSe_2_, Pb doping gives the largest formation energy of 2.19 eV, which is rational considering that cationic substitution should become more facile when the lattice has been distorted. Other factors including entropic contribution and solvation effects might further lower the formation energy, making the formation of M-CoSe_2_ reasonable. Our findings suggest the formation of M-CoSe_2_-DETA is generally facile and the M-CoSe_2_-DETA lattices are thermodynamically stable.

### Synthesis and structural characterization

**Fig. 2:**
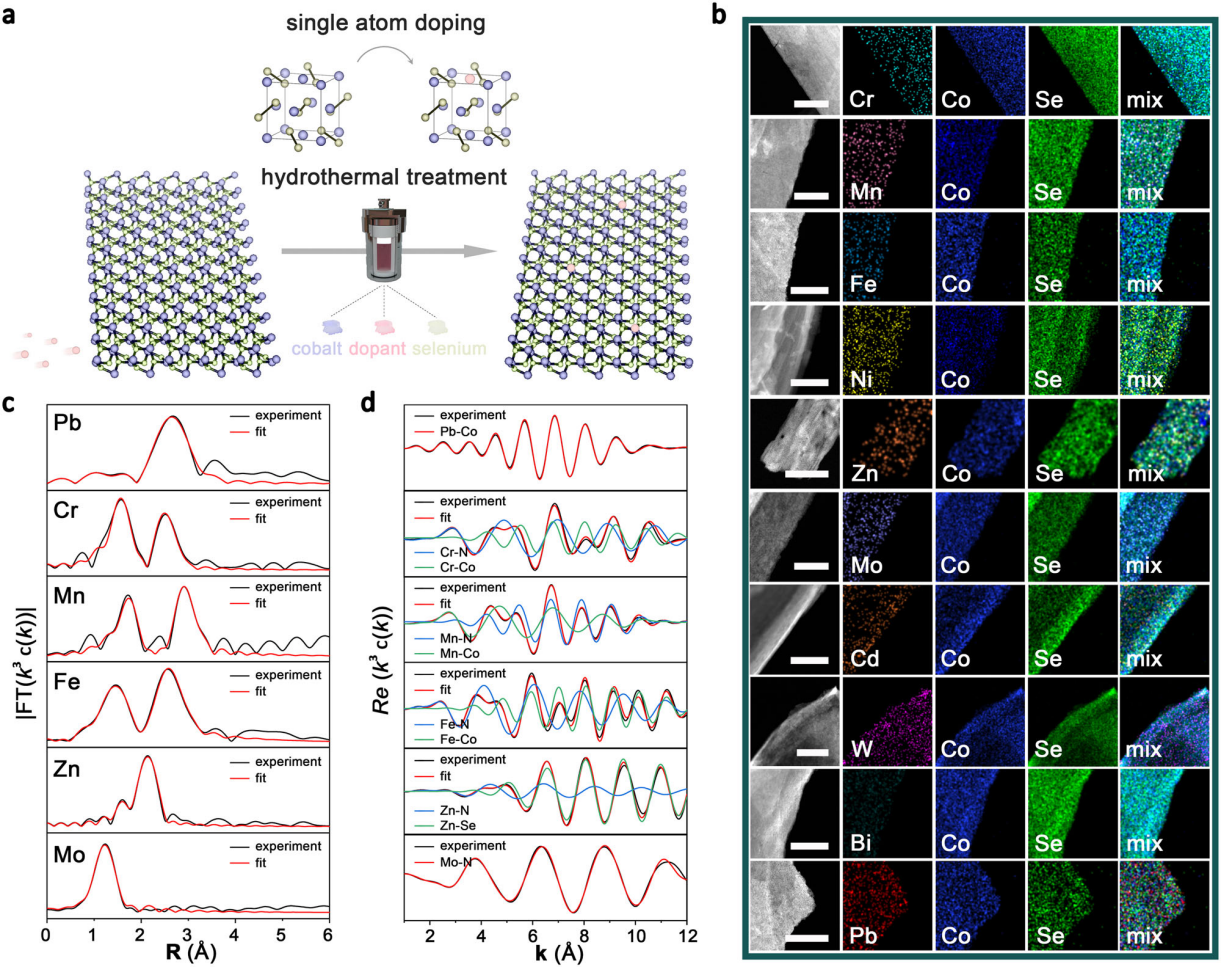
Synthesis and structural analysis. **a** Schematic illustration of the hydrothermal synthesis of M-CoSe_2_-DETA. The balls in purple, green and pink represent Co, Se, and dopants, respectively. **b** STEM elemental mapping images of M-CoSe_2_-DETA nanobelts. Scale bars, 100 nm. **c**, **d R**-space curve-fitting of EXAFS spectra (**c**) and corresponding Re(*k*^3^*χ*(*k*)) oscillations (**d**) of Pb-, Cr-, Mn-, Fe-, Zn- and Mo- CoSe_2_-DETA samples.

Our approach is based on the hydrothermal synthesis of mesostructured CoSe_2_-DETA nanobelts as reported previously^44^. In a typical synthesis, 1 mmol Co(AC)_2_⋅H_2_O and 1 mmol Na_2_SeO_3_ were dissolved in a mixed solution (volume ration of DETA/DIW = 2:1, DIW = deionized water), which was then maintained in a Teflon-lined autoclave at 180 °C for 17 h. To introduce cationic dopants, different precursors were added together with cobalt acetate to prepare M-CoSe_2_-DETA nanobelts (Fig. 2a, see detail in Methods). The molar ratio of dopant/Co is 5:95 with total cationic molar amount of 1 mmol. The crystal structures of M-CoSe_2_-DETA were characterized by X-ray diffraction (XRD). As shown in Supplementary Fig. 1, diffraction peaks of all the as-prepared M-CoSe_2_-DETA show negligible difference from undoped CoSe_2_-DETA, matching well with the cubic phase CoSe_2_ structure^44^ (JCPDS 9-234), suggesting the successful doping of the foreign atoms into the lattice of CoSe_2_ rather than the phase separation^6^.

To shed light on the morphology of M-CoSe_2_-DETA products, we adopted several microscopic characterizations. As confirmed by scanning electron microscopy (SEM) and transmission electron microscopy (TEM) images of M-CoSe_2_-DETA sample, we obtained consistent nanobelt-like morphology with width of hundreds of nanometers and length up to several micrometers, which is similar to that of pure CoSe_2_-DETA sample (Supplementary Fig. 2 to Fig. 12). The high-angle annular dark-field (HAADF) scanning transmission electron microscopy (STEM) elemental mapping images suggest in all doped products, dopants uniformly distribute over the nanobelts (Fig. 2b). The successful introduction of ten cationic dopants is also supported by XPS tests (Supplementary Fig. 13 to Fig. 23). Peaks in dopant regions suggest their existence in products. Furthermore, satellite peaks locate around 780.6 eV and 796.1 eV in Co 2p region, as well as those located around 59.0 eV in Se 3d region, indicate the oxidation of samples in the air. The binding energy mismatch in Co and Se regions between pure CoSe_2_-DETA and M-CoSe_2_-DETA clearly suggests electron transfer caused by cationic doping.

### Local structure of single-atom dopants in M-CoSe_2_-DETA

**Fig. 3:**
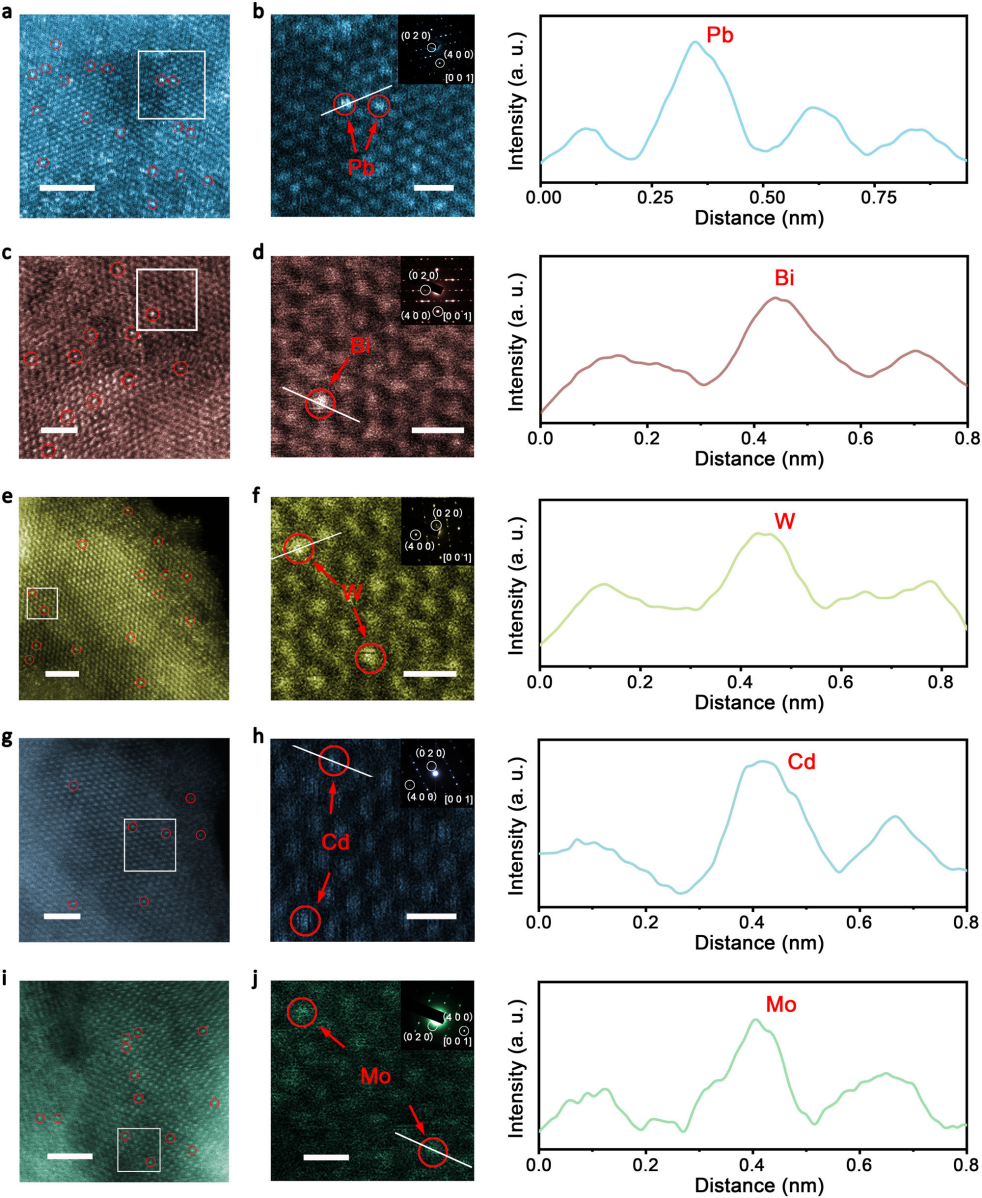
Aberration-corrected STEM images. Atomic-resolution STEM images (**a**, **c**, **e**, **g**, **i**, scale bars, 1 nm) and enlarged images of white rectangles (**b**, **d**, **h**, **f**, **j**, scale bars, 0.5 nm) of Pb-, Bi-, W-, Cd- and Mo-CoSe_2_-DETA nanobelts, respectively. Insets in each enlarged image show corresponding SAED patterns. Right shows intensity profiles along white lines in each enlarged image.

We then investigated the detailed coordination environment of dopants in M-CoSe_2_-DETA samples. We first used aberration-corrected (AC) HAADF-STEM to reveal the precise location of Pb atoms in representative Pb-CoSe_2_-DETA. As displayed in Fig. 3a, the STEM image of a large area suggests the random orientation of Pb-CoSe_2_-DETA, and bright spots (red circles) represent heavy single Pb atoms. We enlarged a rectangle area in Fig. 3a (Fig. 3b), in which two high-density bright spots (highlighted by red circles and arrows in Fig. 3a and b, respectively) can be assigned to Pb single atoms. The selected-area electron diffraction (SAED) patterns (corresponded to rectangle area in Supplementary Fig. 12) clearly demonstrate [0 0 1] orientation (inset in Fig. 3b). Exposed facets, such as those with Miller indices of {4 0 0} and {0 2 0}, can be well recognized (white circles). Using image contrast, the integrated pixel intensities of cations demonstrate each column can be exactly associated with their atomic identities as shown in Fig. 3b. Moreover, we also carried out X-ray absorption near-edge spectroscopy (XANES) and extended X-ray absorption fine structure (EXAFS) at the L-edge of Pb to probe its fine local structure. As shown by the Fourier transform (FT) of *k*_3_-weighted *χ*(*k*)-function of the EXAFS spectra in **R** space in Fig. 2c and corresponding oscillations in Fig. 2d, only one dominant peak exists at ca. 2.66 Å, which could be assigned to Pb-Co bond. According to the fitting results (Supplementary Table 2), coordination number of Pb-Co is about 3.3. The microscopical and XAS analysis confirm Pb as single atom dopant.

In addition to Pb-CoSe_2_-DETA, this hydrothermal strategy also proved available for preparing Bi, W, Cd, Mo, Zn, Ni, Fe, Mn and Cr single-atom doped CoSe_2_-DETA nanobelts. Bright spots are clearly observed in STEM images (red circles, Figs. 3c, e, g and i), demonstrating heavy single atom dopants (Bi, W, Cd and Mo), which can be directly visualized in STEM images, existing in M-CoSe_2_-DETA nanobelts. For Zn-, Ni-, Fe-, Mn- and Cr-CoSe_2_-DETA nanobelts, no obvious bright spots are acquired presumably due to the approximate locations of these elements with Co in the periodic table of elements (Supplement Fig. 30 to Fig. 34). The isolated bright spots in magnified STEM images (Fig. 3d, f, h and j) of rectangle areas in Fig. 3c, e, g and i provide evidence that the single atom dopants substitute Co atoms in CoSe_2_ lattice. Their integrated pixel intensity profiles again confirm the substitution of Co atoms with single heavy metal dopants (Fig. 3d, f, h and j). The SAED patterns (insets in Fig. 3d, f, h and j, corresponded to rectangle areas in Supplementary Fig. 11, Fig. 10, Fig. 9 and Fig. 8) suggest the Miller indices of exposed planes for M-CoSe_2_-DETA are {4 0 0} and {0 2 0} with [0 0 1] ligaments, same as that of Pb-CoSe_2_-DETA (Fig. 3b). We also conducted XAS measurements to investigate their local coordination environment. Several EXAFS best-fitting analysis and fitting results are shown in Fig. 2c, d and Supplementary Table 2. Evidently, predominant peaks can all be assigned to dopant-Co bond, dopant-Se bond and dopant-N bond. No dopant-dopant bond was observed, suggesting the isolated doping atoms in CoSe_2_-DETA lattice. Combine above-mentioned results, the generalizability of this strategy has been demonstrated for synthesizing a library of single-atom doped CoSe_2_-DETA nanobelts.

### Electrochemical HER activity

**Fig. 4:**
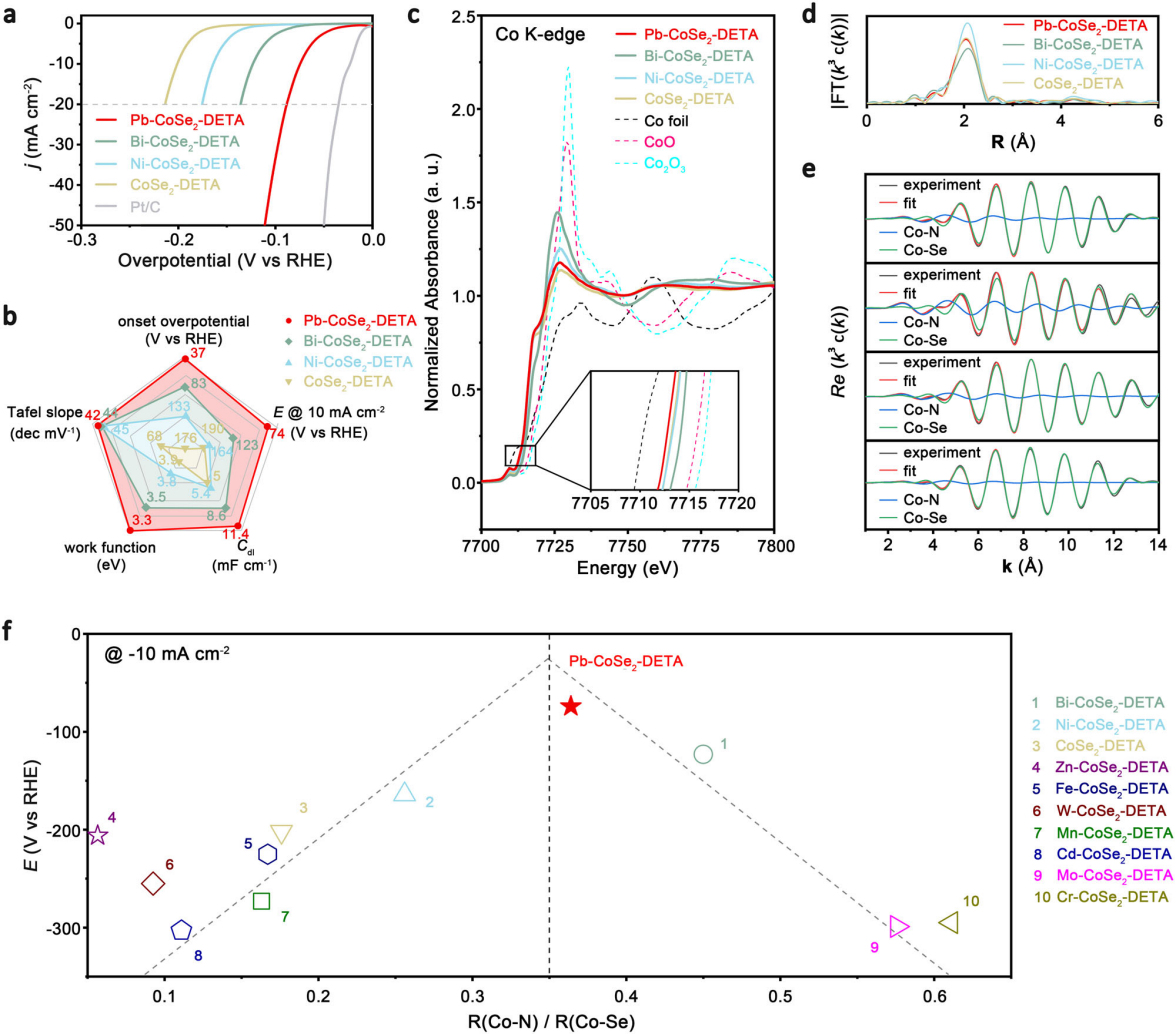
HER performance and activity descriptor. **a** Electrocatalytic HER of Pb-, Bi-, Ni-CoSe_2_-DETA, pure CoSe_2_-DETA and commercial Pt/C in 0.5 M H_2_SO_4_. **b** Activity comparison of Pb-, Bi-, Ni- CoSe_2_-DETA and pure CoSe_2_-DETA. **c** XANES spectra at Co K-edge of Pb-, Bi-, Ni-CoSe_2_-DETA, pure CoSe_2_-DETA, Co foil, CoO and Co_2_O_3_. Inset shows magnified pre-edge region in black rectangle. **d**, **e R**-space curve-fitting of EXAFS spectra (**d**) and corresponding Re(*k*^3^*χ*(*k*)) oscillations (**e**) of Pb-, Bi-, Ni-CoSe_2_-DETA and pure CoSe_2_-DETA samples. **f** Relation between overpotentials at 10 mA cm^−2^ and R value. A dashed volcano line is shown for guidance.

We deposited all products onto glassy carbon rotating-disk electrode (RDE) and tested their electrochemical HER activity in a standard three-electrode system in acidic media (H_2_-saturated 0.5 M H_2_SO_4_ electrolyte, pH ~0.35), with graphite rod counter electrode and Ag/AgCl reference electrode. For comparison, commercial state-of-the-art Pt/C (40 wt%) and undoped CoSe_2_-DETA were also tested under the same condition. Polarization curves suggest that CoSe_2_-DETA shows an onset overpotential of 176 mV and the required overpotential to reach current density of 10 mA/cm^2^ (based on geometric area) is 190 mV, which are similar to previous reports (Fig. 4a)^43^. Incorporation of single atom in CoSe_2_-DETA leads to the negative or positive shifted HER behaviors, which can be ascribed to the different electronic structure caused by different dopants (discussed later). Polarization curves of samples with enhanced HER activity are also represented in Fig. 4a. Among various M-CoSe_2_-DETA samples, Pb-CoSe_2_-DETA performs best, starting catalyzing HER at merely 37 mV; for comparison, onset overpotentials of Bi- and Ni-CoSe_2_-DETA samples are 83 and 133 mV. The required overpotential to achieve current density of 10 mA/cm^2^ is as low as 74 mV for Pb-CoSe_2_-DETA, outperforming that of 123 mV for Bi-CoSe_2_-DETA and 164 mV for Ni-CoSe_2_-DETA (Fig. 4b), suggesting energetical merits of Pb-CoSe_2_-DETA catalyst. Tafel analysis gives Tafel slopes of merely 42, 44 and 45 mV/dec for Pb-, Bi- and Ni-CoSe_2_-DETA, respectively, which are much smaller than that of 68 mV/dec for CoSe_2_-DETA, suggesting their much improved HER kinetics. These Tafel slope values also hint to a Heyrovsky-Volmer pathway with Heyrovsky rate-limiting step.

We also recorded electrochemical impedance spectroscopy (EIS) to probe charge transfer resistance (*R*_ct_) at 74 mV (10 mA/cm^2^ for Pb-CoSe_2_-DETA; Supplementary Fig. 39). The recorded *R*_ct_ of Pb-CoSe_2_-DETA is 4.8 Ω, vs. 8.1 Ω for Bi-CoSe_2_-DETA, 11.8 Ω for Ni-CoSe_2_-DETA and 19.7 Ω for CoSe_2_-DETA. The smallest *R*_ct_ value for Pb-CoSe_2_-DETA indicates much improved charge transfer kinetics. To elucidate the effect of real surface area modulation caused by different dopants, we measured electrochemically active surface areas (ECSAs) of products using double-layer capacitance (*C*_dl_). We found the *C*_dl_ value of Pb-CoSe_2_-DETA (11.4 mF cm^−2^) is 2.28 times higher than that of CoSe_2_-DETA (5.0 mF cm^−2^), showing great enhancement compared with those of Bi- and Ni-CoSe_2_-DETA (8.6 and 5.4 mF cm^−2^, respectively; Fig. 4b). This demonstrates more accessible HER active sites created on Pb-CoSe_2_-DETA. We then measured work functions to estimate electron transfer rate and chemical reactivity. It’s no surprising that Pb-CoSe_2_-DETA has the lowest work function of 3.3 eV. All above energetic and kinetic metrics, including onset overpotential, Tafel slope, *C*_dl_ and charge transfer rate, suggest Pb atom as the best modulator to make HER activity of Pb-CoSe_2_-DETA far exceeding other doped CoSe_2_-DETA samples.

The long-term stability is another essential parameter to appraise catalysts. Accelerated voltammograms (CVs) cycling tests evidence the robustness of catalysts with identical polarization curves before and after 1000 CVs cycles (Supplementary Fig. 40). We then performed chronoamperometry tests, running water reduction on studied catalysts deposited on carbon fiber paper (CPF) under constant current density of 10 mA/cm^2^. No obvious changes in overpotential were observed over 20 h continuous operation (Supplementary Fig. 41). Postmortem STEM images after stability tests demonstrate the nanobelt-like morphology can be well maintained with uniform distribution of dopants, Co and Se (Supplementary Fig. 42 to Fig. 44). XPS spectra of the cycled samples reveal that the valance states of elements have no change after long-term stability tests (Supplementary Fig. 45 to Fig. 47). The above results unambiguously illustrate their excellent long-term HER stability in acidic electrolyte.

### Identification of HER activity descriptor

We now aim to identify an HER activity descriptor related to the intrinsic electronic structure of catalysts. We began by conducting XAS at the K edge of Co to analyze their fine local structure. Typical EXANE spectra of Pb-, Bi-, Ni-CoSe_2_-DETA and CoSe_2_-DETA samples, along with Co foil, CoO and Co_2_O_3_ with references, are presented in Fig. 4c. The absorption edges of Pb-, Bi-, Ni-CoSe_2_-DETA and CoSe_2_-DETA samples lays between Co foil and CoO, suggesting Co oxidation states between 0 and +2. To further analyze the local atomic structure, we show their FT of *k*_3_-weighted *χ*(*k*)-function of Co EXAFS spectra in **R** space as well as EXAFS best-fitting curves (Fig. 4d and e, Supplementary Fig. 49 and Fig. 50). All samples display similar oscillation frequencies, with a major peak at about ca. 2.1 Å. We quantitatively carried out least-squares EXAFS curve-fitting analysis of Co by considering two backscattering paths, Co–N and Co–Se. The best-fitting analysis (Fig. 4e and Supplementary Table 3) clearly reveal the major peak originates from Co-N bonds (about ca. 2.01 Å) and Co-Se bonds (about ca. 2.40 Å) with different coordination numbers. For example, coordination numbers of Co-N bonds, R(Co-N), and Co-Se, R(Co-Se), bonds for Pb-CoSe_2_-DETA are 1.4 and 4.4, respectively, while those for Ni-CoSe_2_-DETA are 1.1 and 4.3, respectively. The fitted structural results demonstrate different dopants can tune the local atomic structure of Co atoms in CoSe_2_ lattice. This is reasonable as dopants will introduce heterogeneous spin states and cause subtle distortion of pristine lattice^48^. The distortion originates from: (1) the localized Coulomb interaction around dopants with incoordinate electron spins; (2) the electron configuration of Co^2+^ is *t*_2g_^6^*e*_g_^1^ with strong Jahn–Teller effect, so that dopants with no Jahn–Teller effect (such as Ni^2+^, *t*_2g_^6^*e*_g_^2^) well lead to mismatch in the degree of Jahn–Teller distortion; (3) dopants with larger atomic radius, such as Pb and Mo, will probably squeeze or even occupy sites of neighboring Co or Se atoms, which is consistent with the totally different EXAFS best-fitting analysis (Fig. 2c and d).

The previous studies have confirmed Co as HER active sites in CoSe_2_^41,43^. This is because that 3*d* states of Co hybridize with Se to form *e*_g_ orbital in CoSe_2_ structure, which primarily contributes to the Fermi level. Here we demonstrate HER activity of all doped CoSe_2_-DETA exhibits a volcano-shape plot as a function of Co local structure (Fig. 4f). Co local structure mentioned here manifests changing of ratios of Co-N bonds to Co-Se bonds (R, calculated from XAS data mentioned above), and HER activities are in terms of overpotentials required to achieve current density of 10 mA/cm^2^. Among 10 cationic dopants, Pb-CoSe_2_-DETA locates most close to the top of the “volcano”. To understand origin HER energetics associated with R, we performed DFT calculations to study the electronic structure of these catalysts. Note that changing dopants can lead to large alteration of R values. For simplicity, we constructed the structure models based on pure CoSe_2_ with controllable amount of -NH_2_ on the surface, which therefore represent catalysts with different R values. The calculated projected density of states (PDOS) proves that catalyst with R of 0.35 possess the highest electronic states near the Fermi level, demonstrating its promoted electron transfer rate and high conductivity which can facilitate HER process (Supplementary Fig. 52). With increased or decreased R, electronic states near the Fermi level all decrease. We note that, on the basis of diffraction shift shown in Supplementary Fig. 1, the lattice parameter-HER activity dependency can also be established to analyze the relationship (Supplementary Fig. 53, Supplementary Table 5)^49^.

**Fig. 5:**
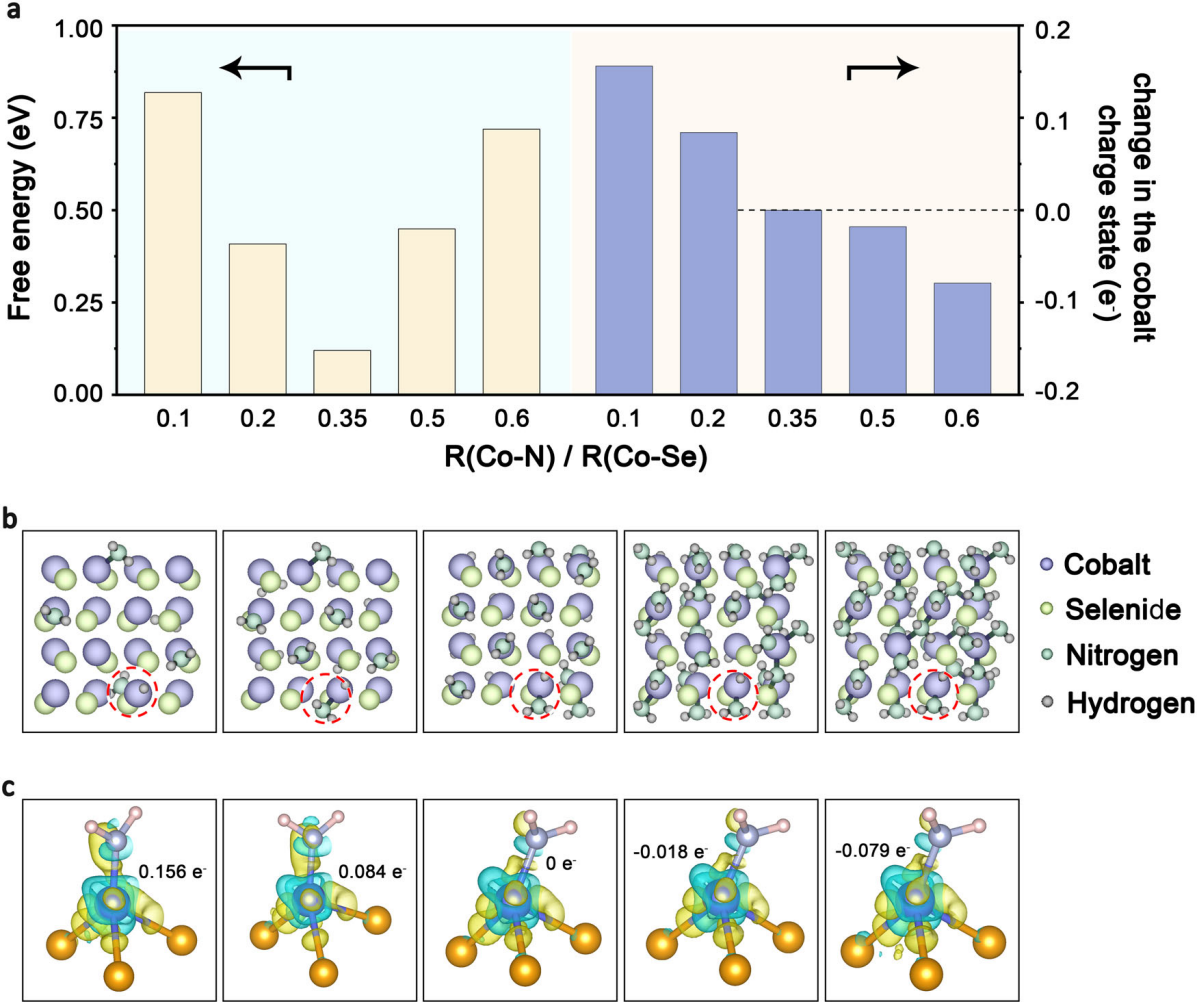
HER DFT calculations. **a** Calculated free energies and cobalt charge state changes of different R values, the ratio of number of Co-N bonds (R(Co-N)) to number of Co-Se bonds (R(Co-Se)). **b**, **c** Crystal structures (**b**, the balls in purple, green, dark green and gray represent Co, Se, N and H, respectively) and differential charge densities (**c**, the balls in purple, orange, dark blue and pink represent Co, Se, N and H, respectively) of studied model with R values of 0.1, 0.2, 0.35, 0.5 and 0.6 (from left to right).

We also conclude such relationship between HER activity and Co local structure with support by molecular orbital theory. When H atom absorbs on surface Co atom, H 1 *s* orbital will combine with Co 3*d* orbital to form bonding and anti-bonding orbitals, and the energy level mismatching between H 1 *s* orbital and Co 3*d* orbital determines H-Co bonding strength (Supplementary Fig. 54). As confirmed by the Bader charge of Co atoms (Fig. 5a, c and Supplementary Table 4), the value decreases with increasing of R. That is to say, cationic dopants can modulate Co local structure and lead to the change of electronic charge density of Co atom, thus tuning energy level mismatching and modifying HER activity (Supplementary Fig. 42). To quantitively evaluate the relationship between R and HER activity, we then calculated Δ*G*_H_ values on catalytic sites. As shown in Fig. 5a, |Δ*G*_H_ | first decreases with increasing of R. When R is larger than 0.35, increase of R will lead to increase of | Δ*G*_H_ | . The theoretical volcano-shape relationship here is similar to experimental data (Fig. 4f). Specifically, for calculated models, we obtained the lowest | Δ*G*_H_ | value (0.12 eV) at the model with *R* = 0.35, which is very close to optimal Pb-CoSe_2_-DETA samples (*R* = 0.364). These results rationalize HER energetics caused by different R.

## Discussion

In summary, we report a facile method to prepare a library of single-atom doped CoSe_2_-DETA nanobelts consisting of 10 compounds. This general single-atom doping strategy is strongly convinced by STEM and XAS studies, resulting in the modulation of Co local coordination as well as the change of HER performance. We conclude a volcano-shape relationship between HER activity and the ratio of Co-N bonds to Co-Se bonds, with Pb-CoSe_2_-DETA performing the best HER activity. Our findings suggest a rational design principle for CoSe_2_-based SADCs with enhanced HER activity by modulating coordination environment of Co using suitable single atom dopants. Motivated by above-mentioned results, such a single-atom doping induced atomic configuration-regulating strategy may act as a promising approach to efficient non-noble-metal based catalysts in energy conversion and storage devices.

## Methods

### Materials synthesis

CoSe_2_-DETA nanobelts in this study are synthesized as previously reported^44^. Co(AC)_2_⋅4H_2_O, Na_2_SeO_3_, and DETA were purchasing from Sinopharm Chemical Reagent Co., Ltd.. In a typical synthesis, 1 mmol Co(AC)_2_⋅4H_2_O was added into a mixed solution of DETA and DIW (total volume, 40 mL; volume ratio of V_DETA_/V_DIW_ = 2:1; DIW = deionized water). Then 1 mmol Na_2_SeO_3_ was added under stirring. After stirring for half an hour, the solution was transferred into a Teflon-lined autoclave, maintaining at 180 °C for 17 h. It was then cooled to room temperature naturally. The resulting black products were washed with absolute ethanol five times and dried under vacuum at 60 °C for 12 h. For the synthesis of M-CoSe_2_-DETA nanobelts, the whole procedure was the same except 1 mmol Co(AC)_2_⋅4H_2_O was changed into 0.095 mmol Co(AC)_2_⋅4H_2_O and 0.005 mmol doped cationic precursors, including Cr_3_(OH)_2_(AC)_7_, Mn(AC)_2_⋅5H_2_O, Fe(AC)_3_, Ni(AC)_2_⋅4H_2_O, Zn(AC)_2_⋅2H_2_O, Cd(AC)_2_⋅2H_2_O, Bi(NO_3_)_2_⋅5H_2_O, Pb(AC)_2_⋅3H_2_O, purchasing from Sinopharm Chemical Reagent Co., Ltd., and C_10_H_14_MoO_6_, WCl_6_, purchasing from Sigma Aldrich.

### Characterizations

The products were examined by various analytic techniques. Scanning electron microscope (SEM, JSM-6700F) was applied to investigate the size and morphology. X-Ray powder diffraction (XRD) was carried out on a Bruker D8 X-ray diffractometer with Cu Ka radiation (λ = 1.5406 Å) and a scintillation counter. The morphology of the as-synthesized samples was determined by using a JEOL 2010F(s) transmission electron microscope (TEM). The high-resolution TEM (HRTEM) observation and selected-area electron diffraction (SAED) were taken on the same machine with an acceleration voltage of 200 kV. High-angle annular dark-field scanning TEM (HAADF-STEM) images and the corresponding energy dispersive X-ray (EDX) spectra elemental mapping were recorded on a FEI Titan Cubed Themis G2 300 with a probe corrector at 200 kV. ICP data were obtained by an Optima 7300 DV instrument. The X-ray photoelectron spectra (XPS) and Ultraviolet-photoelectron spectroscopy (UPS) were performed at the Catalysis and Surface Science Endstation at the BL11U beamline in the National Synchrotron Radiation Laboratory (NSRL) in Hefei, China. The X-ray absorption edge spectra were collected on BL14W1 beamline of Shanghai Synchrotron Radiation Facility (SSRF) and analyzed with software of Ifeffit Athena.

### Electrochemical measurements

Electrochemical measurements were performed at room temperature using a typical three-electrode system, connected to a Multipotentiostat (IM6ex, ZAHNER elektrik, Germany), with graphite rod as the counter electrode and Ag/AgCl (in saturated aq. KCl) as the reference electrode, respectively, in 0.5 M H_2_SO_4_ (pH ~0.35) electrolyte. To make working electrodes, 5 mg catalyst powder was dispersed in 1 mL of 1:2 v/v isopropanol/DIW mixture with 50 μL Nafion solution (5 wt%), which was ultrasonicated to yield a homogeneous ink. Then, 50 μL ink was drop-cast onto the glassy carbon electrode (catalyst loading: 1.21 mg cm^−2^). Before the electrochemical HER measurement, the electrolyte was degassed by bubbling pure hydrogen for 1 h to ensure the H_2_-saturation of the electrolyte, respectively.

The polarization curves were obtained by sweeping the potential with a rate of 5 mV s^−1^. The polarization curves were replotted as overpotential (*η*) vs. log current (log *j*) to get Tafel plots to assess HER kinetics of catalysts. By fitting the linear portion of the Tafel plots to the Tafel equation (*η* = b log (*j*) + a), the Tafel slope (b) is obtained. For comparison, commercial Pt/C (40 wt%, Sigma Aldrich) was measured under the same condition. The cyclic voltammetry (CV) plots were swept between 0 V and −0.15 V. The current density-time (i-t) curves were measured at constant potentials to achieve current density of −10 mA cm^−2^. Electrochemical impedance spectroscopy (EIS) data of all electrocatalysts for HER and OER were collected from 10,000 to 0.01 Hz at an amplitude of 5 mV with applied overpotentials of −0.074 V, under which the current density of Pb-CoSe_2_-DETA was −10 mA cm^−2^. CVs for double-layer capacitance were determined in the potential window nearly without Faradic process with scan rate of 20, 40, 60, 80, 100, 120, 140 and 160 mV/s.

### *IR* correction

To correct the ohmic drop, all measured potentials were calibrated using equation *E*_cor_ = *E* – *iR*, where *E*_cor_ was corrected potential, *E* was measured potential, *i* was current and *R* was the contact resistance derived from EIS data.

#### DFT calculations

##### Computational methods related to formation energy of doped materials

Periodic, spin-polarized density functional theory (DFT) calculations were done using Vienna Ab initio Simulation Package (VASP), at the level of generalized gradient approximation (GGA) with the Perdew-Wang (PW91) exchange-correlation functional. The projector-augmented wave method (PAW) was used to describe the core electrons, and the Kohn-Sham valence states were expanded in a plane wave basis set with a kinetic energy cutoff of 400 eV.

Only substitutional defect was considered, based on STEM measurements. The substitutional defect formation energy was calculated as Δ*E*_f_ = *E*_d-CoSe2_ – *E*_CoSe2_ + *E*_Co_ – *E*_M_, where *E*_d-CoSe2_, *E*_CoSe2_, *E*_Co_ and *E*_M_ refer to DFT-calculated total energies for doped c-CoSe_2_, undoped c-CoSe_2_, bulk Co and bulk dopant metal, respectively. The equilibrium lattice constants for Co (hcp), Pb (fcc), Ni (fcc), W (bcc), Cr (bcc) and Mo (bcc) were calculated to be 2.493 Å, 5.062 Å, 3.522 Å, 3.176 Å, 2.836 Å and 3.152 Å, respectively, in close agreement with corresponding experimental values (Wyckoff, R. W. G.). A 2 × 2 × 2 unit cell was used to simulate the bulk c-CoSe_2_, with a 6 × 6 × 6 Monkhorst-Pack k-point grid. This corresponds to 32 Co atoms and 64 Se atoms per unit cell for the case of undoped c-CoSe_2_. Given one dopant metal atom per unit cell for the doped systems, a defect concentration of slightly above 3 at% is therefore assumed. The DFT-calculated defect formation energies were summarized in Supplementary Table 1.

##### HER DFT calculations

HER DFT calculations were performed using the plane-wave code Vienna ab initio simulation package (VASP) program with PAW method. The convergence criterion of the electronic self-consistent iteration was set to be 10^−6^ eV and the kinetic energy cutoff is 400 eV. The atomic positions were relaxed until the force on each atom is below 0.005 eV Å^−1^. The Perdew-Burke-Ernzerhof (PBE) GGA exchange-correlation functional was used throughout. A (3 × 3 × 1) Monkhorst-Pack k-grid scheme was used for the calculations of CoSe_2_ with different absorbed -NH_2_. The atomic positions and lattice constants of CoSe_2_ are all optimized. The optimized bulk cell of CoSe_2_ is a = b = c = 5.828 Å.

### Reporting summary

Further information on research design is available in the Nature Portfolio Reporting Summary linked to this article.

## Supplementary information

Supplementary Information

Reporting Summary

## Data Availability

The data that support the findings of this study are available on request from the corresponding author (S.H.Y.).
