# Supplementary material for: Dopant triggered atomic configuration activates water splitting to hydrogen
Source: Nat Commun. 2023 Apr 21;14:2306. doi: 10.1038/s41467-023-37641-3 (PMC10121564; doi:10.1038/s41467-023-37641-3)
Supplement: Supplementary file 1 — Supplementary Information [file 41467_2023_37641_MOESM1_ESM.pdf]

Supplementary Information

Wu et al.

Supplementary Figures

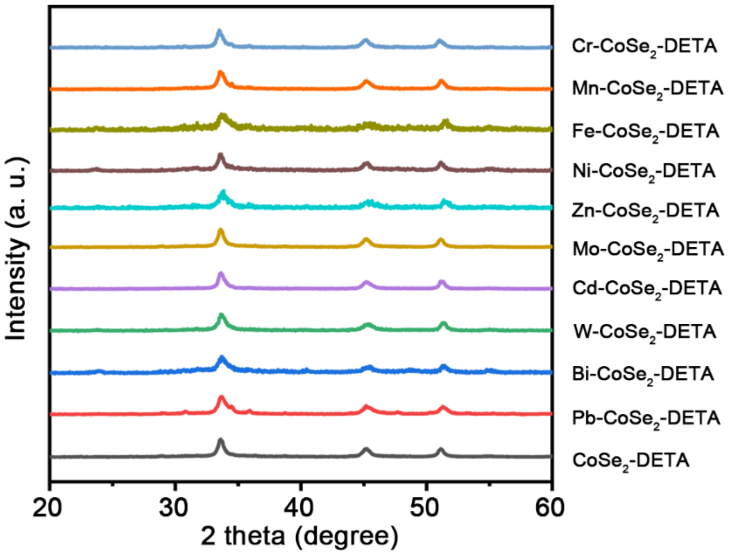

Supplementary Figure 1. XRD patterns of 10 single-atom doped and undoped $CoSe_2$-DETA nanobelts. From bottom to up: $CoSe_2$-DETA, Cr-$CoSe_2$-DETA, Mn-$CoSe_2$-DETA, Fe-$CoSe_2$-DETA, Ni-$CoSe_2$-DETA, Zn-$CoSe_2$-DETA, Mo-$CoSe_2$-DETA, Cd-$CoSe_2$-DETA, W-$CoSe_2$-DETA, Bi-$CoSe_2$-DETA, Pb-$CoSe_2$-DETA.

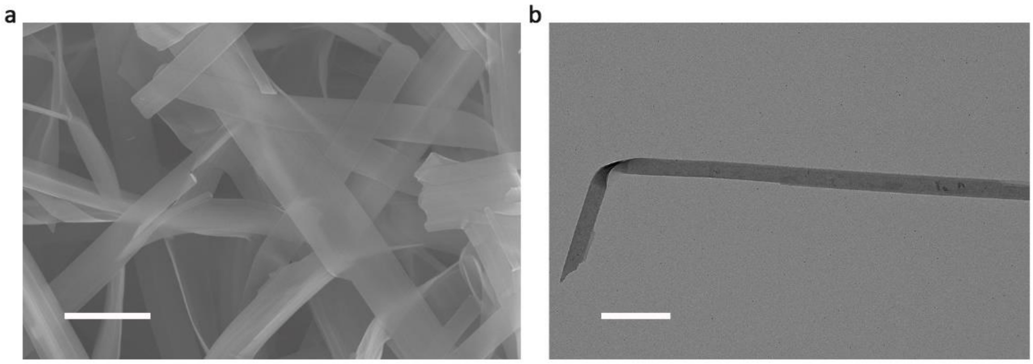

Supplementary Figure 2. Microscopic images. SEM (**a**) and TEM (**b**) images of $CoSe_2$-DETA nanobelts. Scale bar, 200 nm.

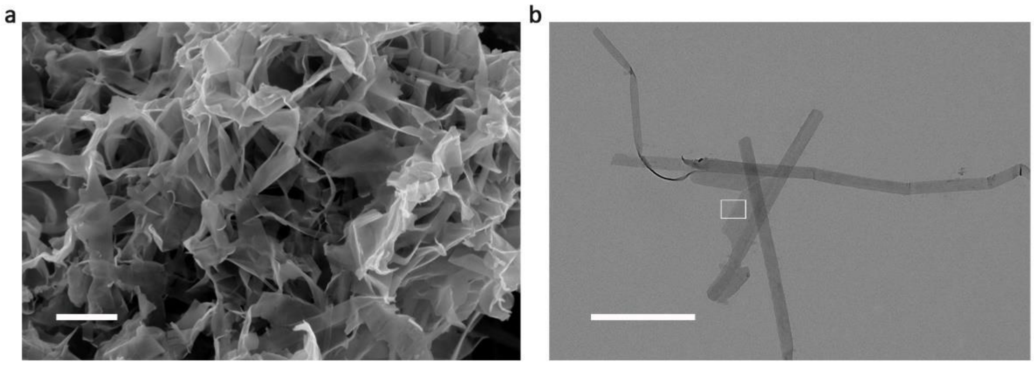

Supplementary Figure 3. Microscopic images. SEM (**a**) and TEM (**b**) images of Cr-$CoSe_2$-DETA nanobelts. Scale bar, 500 nm.

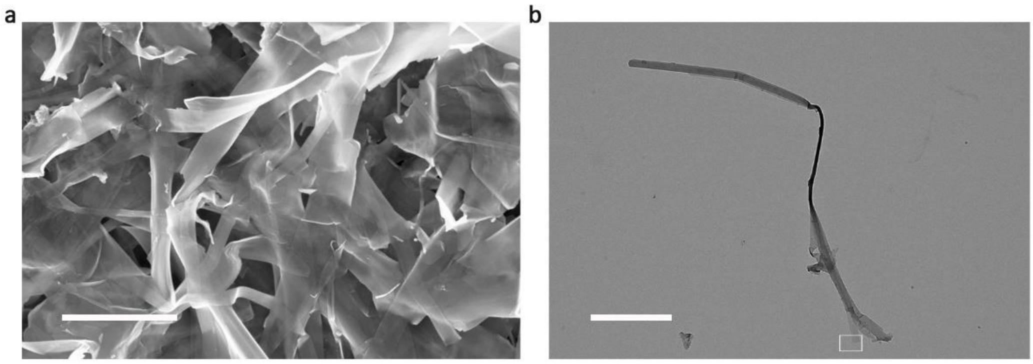

Supplementary Figure 4. Microscopic images. SEM (**a**) and TEM (**b**) images of Mn-$CoSe_2$-DETA nanobelts. Scale bar, 500 nm.

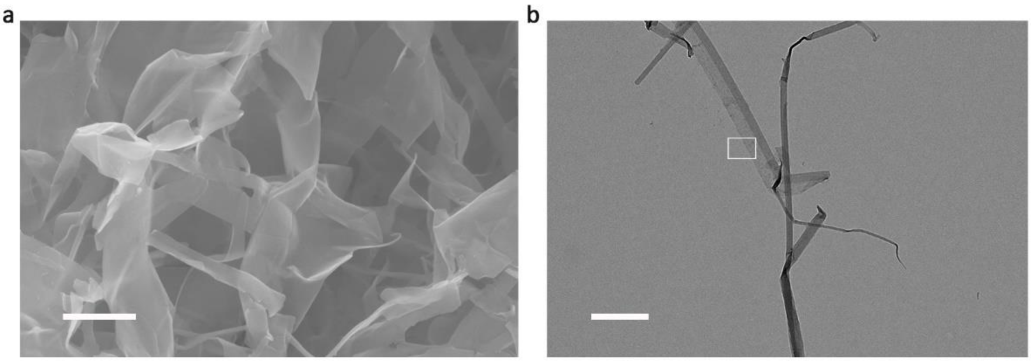

Supplementary Figure 5. Microscopic images. SEM (**a**) and TEM (**b**) images of Fe-$CoSe_2$-DETA nanobelts. Scale bar, 500 nm.

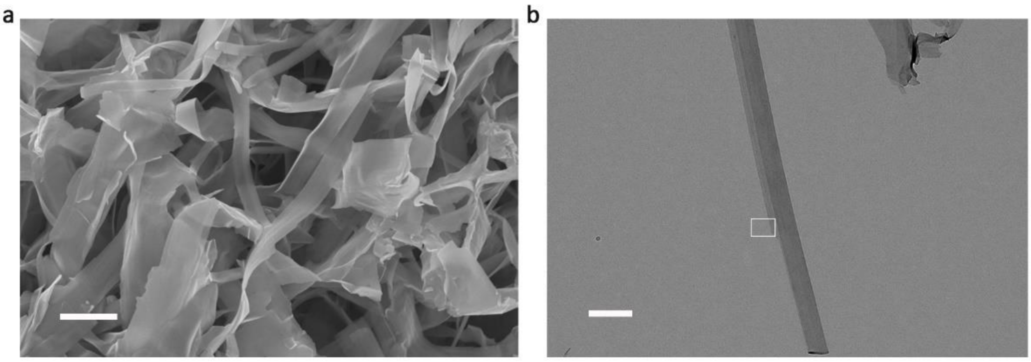

Supplementary Figure 6. Microscopic images. SEM (**a**) and TEM (**b**) images of Ni-$CoSe_2$-DETA nanobelts. Scale bar, 500 nm.

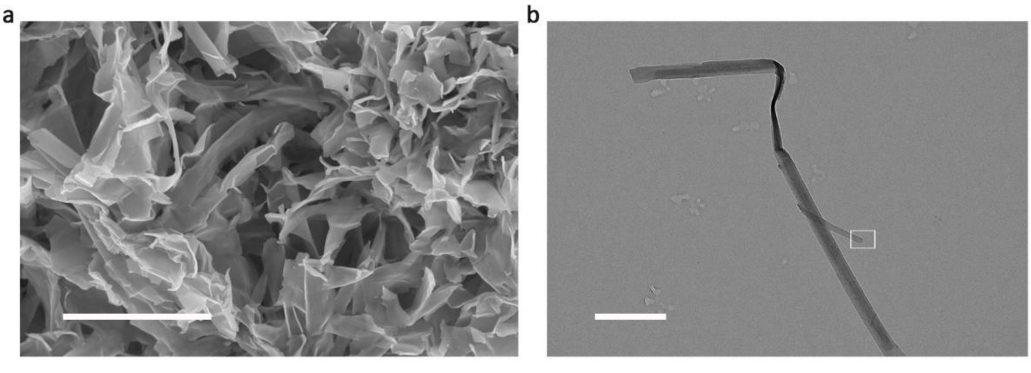

Supplementary Figure 7. Microscopic images. SEM (**a**) and TEM (**b**) images of Zn-$CoSe_2$-DETA nanobelts. Scale bar, 500 nm.

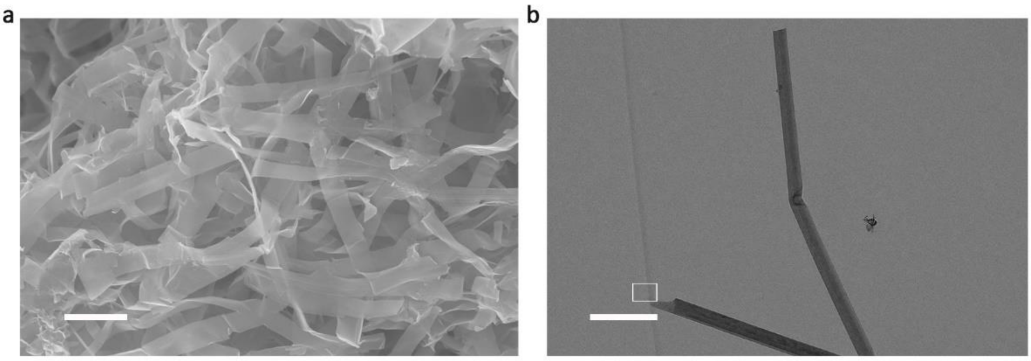

Supplementary Figure 8. Microscopic images. SEM (**a**) and TEM (**b**) images of Mo-$CoSe_2$-DETA nanobelts. Scale bar, 200 nm.

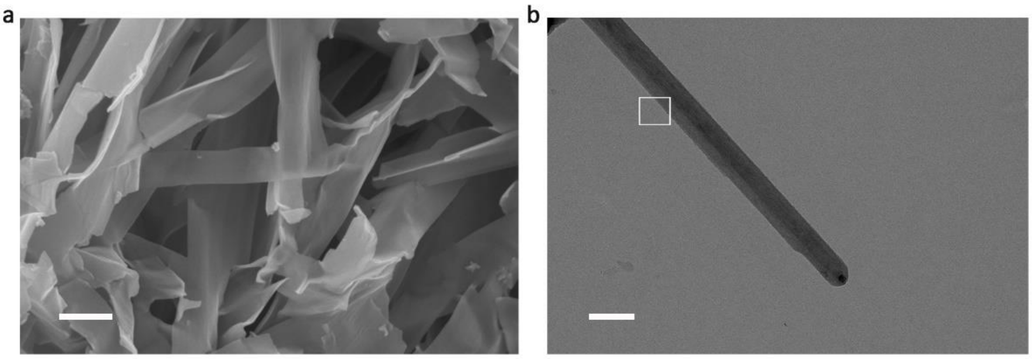

Supplementary Figure 9. Microscopic images. SEM (**a**) and TEM (**b**) images of Cd-$CoSe_2$-DETA nanobelts. Scale bar, 200 nm.

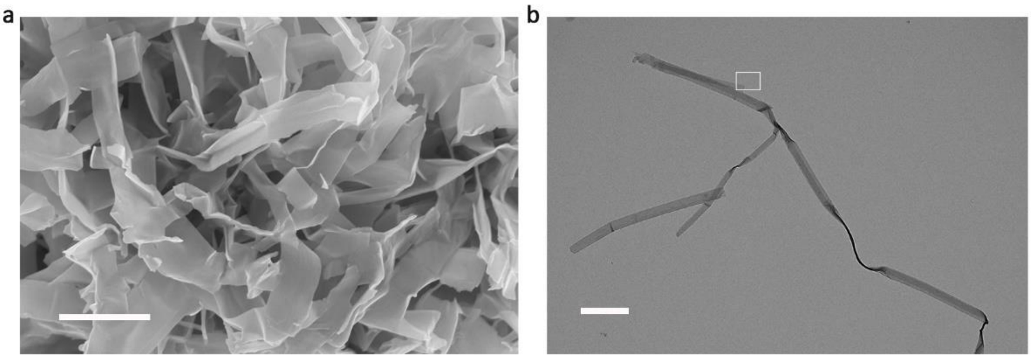

Supplementary Figure 10. Microscopic images. SEM (**a**) and TEM (**b**) images of W-$CoSe_2$-DETA nanobelts. Scale bar, 500 nm.

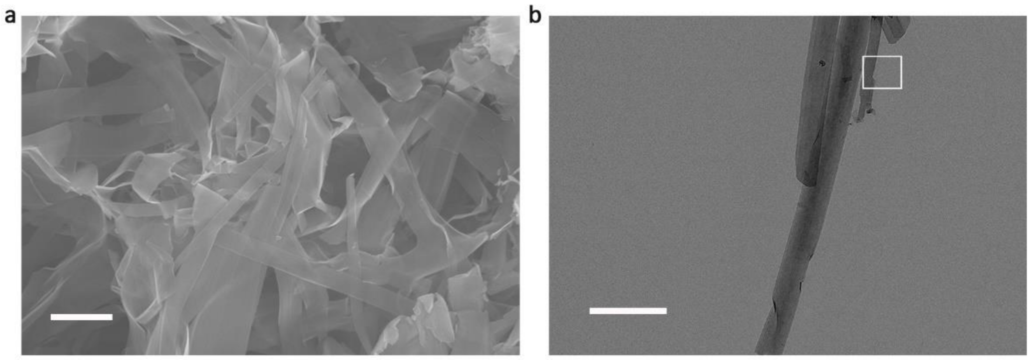

Supplementary Figure 11. Microscopic images. SEM (**a**) and TEM (**b**) images of Bi-$CoSe_2$-DETA nanobelts. Scale bar, 500 nm.

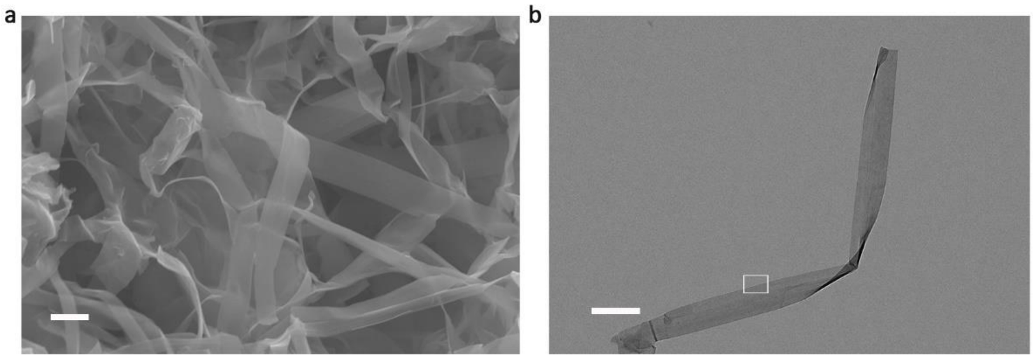

Supplementary Figure 12. Microscopic images. SEM (**a**) and TEM (**b**) images of Pb-$CoSe_2$-DETA nanobelts. Scale bar, 200 nm.

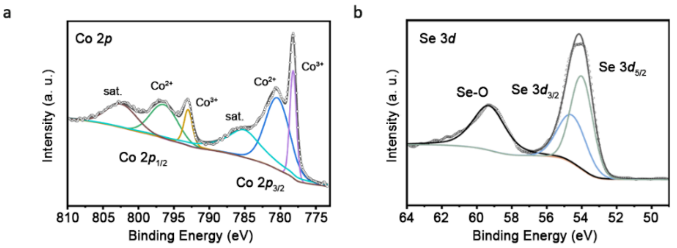

Supplementary Figure 13. XPS spectra. Co 2*p* spectra (**a**) and Se 3*d* spectra (**b**) of $CoSe_2$-DETA.

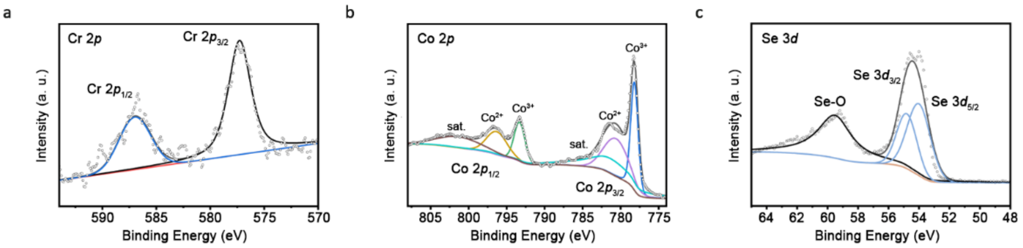

Supplementary Figure 14. XPS spectra. Cr 2*p* spectra (**a**), Co 2*p* spectra (**b**) and Se 3*d* spectra (**c**) of Cr-$CoSe_2$-DETA.

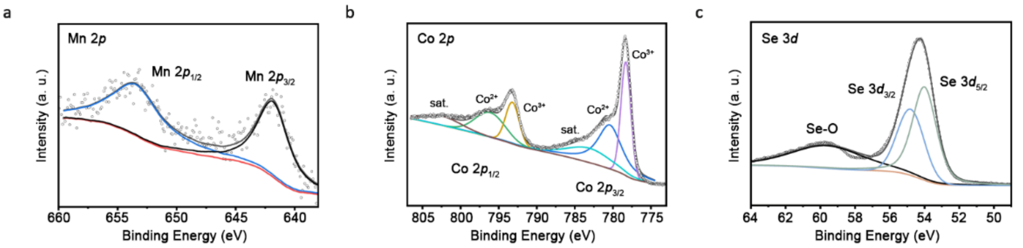

Supplementary Figure 15. XPS spectra. Mn 2*p* spectra (**a**), Co 2*p* spectra (**b**) and Se 3*d* spectra (**c**) of Mn-$CoSe_2$-DETA.

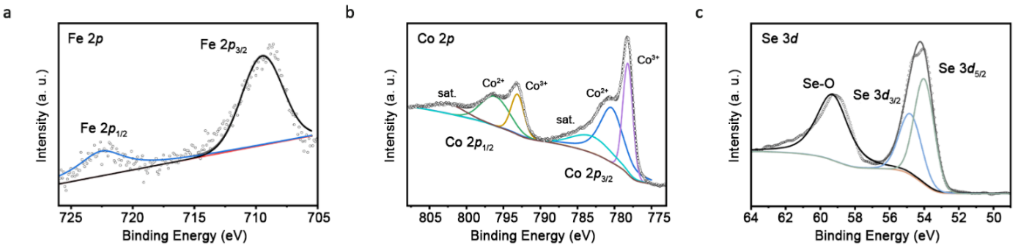

Supplementary Figure 16. XPS spectra. Fe 2*p* spectra (**a**), Co 2*p* spectra (**b**) and Se 3*d* spectra (**c**) of Fe-$CoSe_2$-DETA.

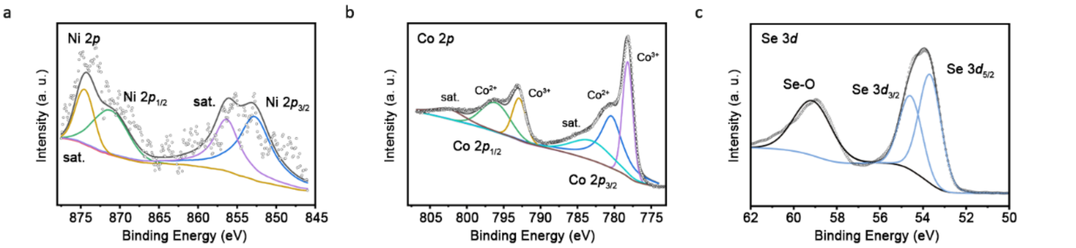

Supplementary Figure 17. XPS spectra. Ni 2*p* spectra (**a**), Co 2*p* spectra (**b**) and Se 3*d* spectra (**c**) of Ni-$CoSe_2$-DETA.

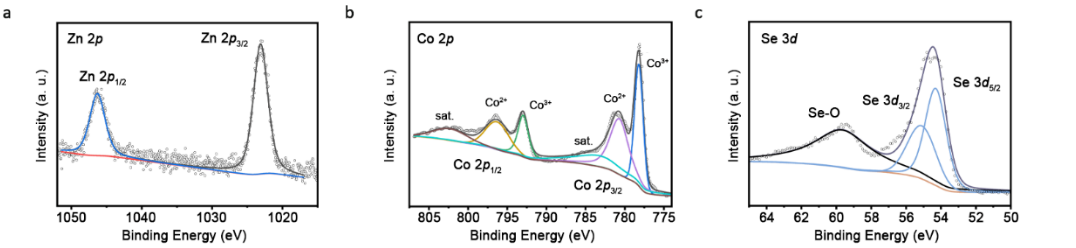

Supplementary Figure 18. XPS spectra. Zn 2*p* spectra (**a**), Co 2*p* spectra (**b**) and Se 3*d* spectra (**c**) of Zn-$CoSe_2$-DETA.

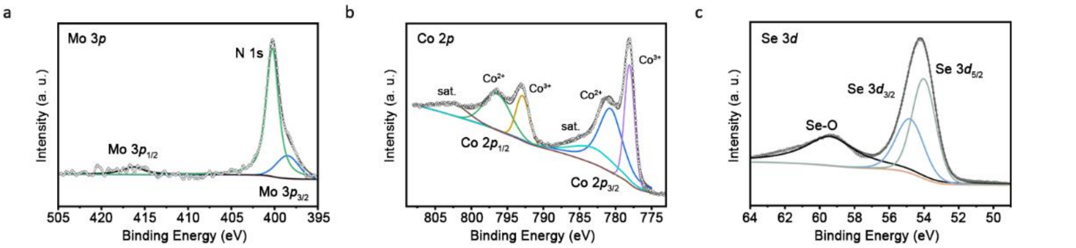

Supplementary Figure 19. XPS spectra. Mo 3*p* spectra (**a**), Co 2*p* spectra (**b**) and Se 3*d* spectra (**c**) of Mo-$CoSe_2$-DETA.

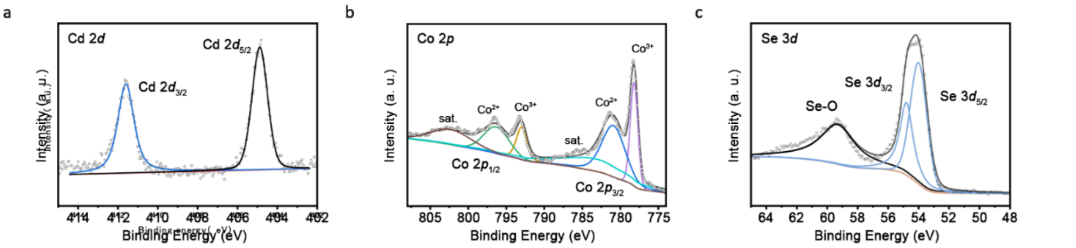

Supplementary Figure 20. XPS spectra. Cd 3*d* spectra (**a**), Co 2*p* spectra (**b**) and Se 3*d* spectra (**c**) of Cd-$CoSe_2$-DETA.

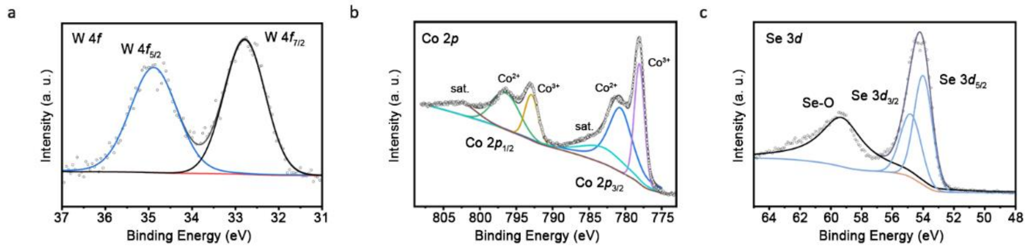

Supplementary Figure 21. XPS spectra. W 4*f* and 5*p* spectra (**a**), Co 2*p* spectra (**b**) and Se 3*d* spectra (**c**) of W-$CoSe_2$-DETA.

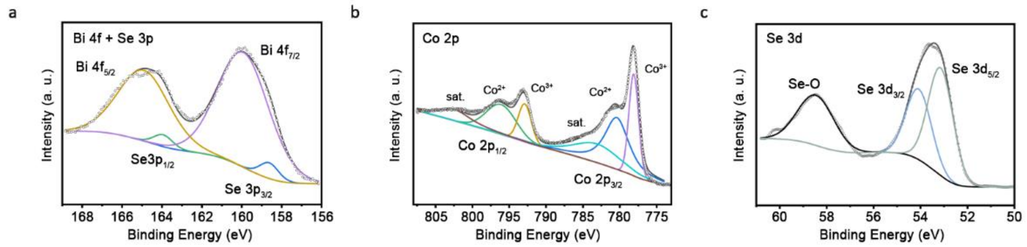

Supplementary Figure 22. XPS spectra. Bi 5*d* spectra (**a**), Co 2*p* spectra (**b**) and Se 3*d* spectra (**c**) of Bi-$CoSe_2$-DETA.

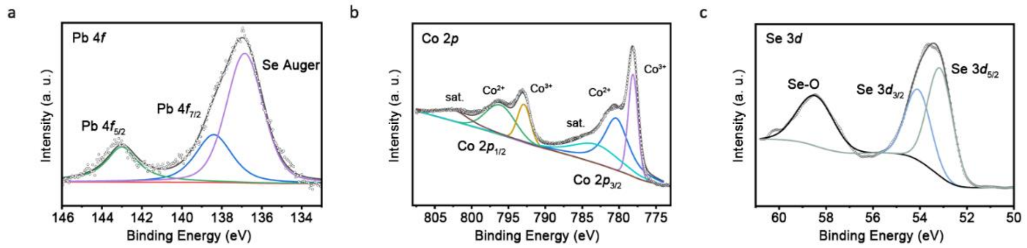

Supplementary Figure 23. XPS spectra. Pb 4*f* spectra (**a**), Co 2*p* spectra (**b**) and Se 3*d* spectra (**c**) of Pb-$CoSe_2$-DETA.

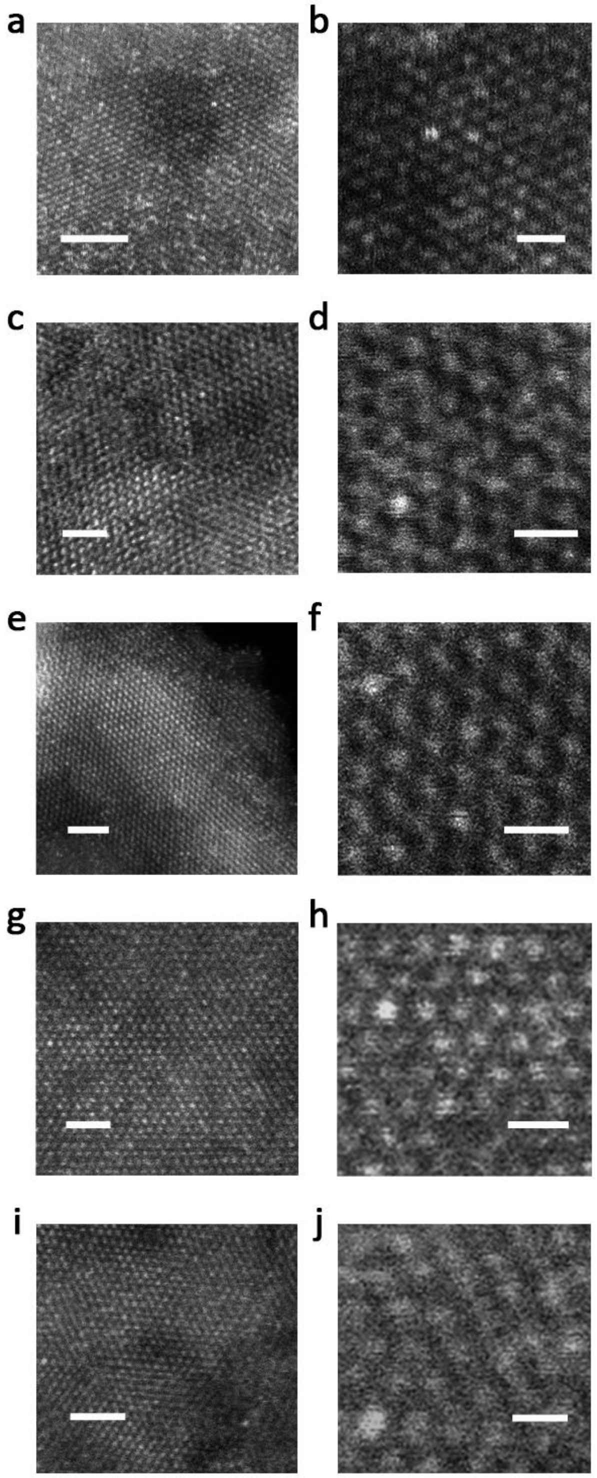

Supplementary Figure 24. Aberration-corrected STEM images. Atomic-resolution STEM images (**a, c, e, g, i**, scale bar, 1 nm) and enlarged images (**b, d, h, f, j**, scale bar, 0.5 nm) of Pb-, Bi-, W-, Cd- and Mo-$CoSe_2$-DETA nanobelts, respectively. All images are original without any post processing.

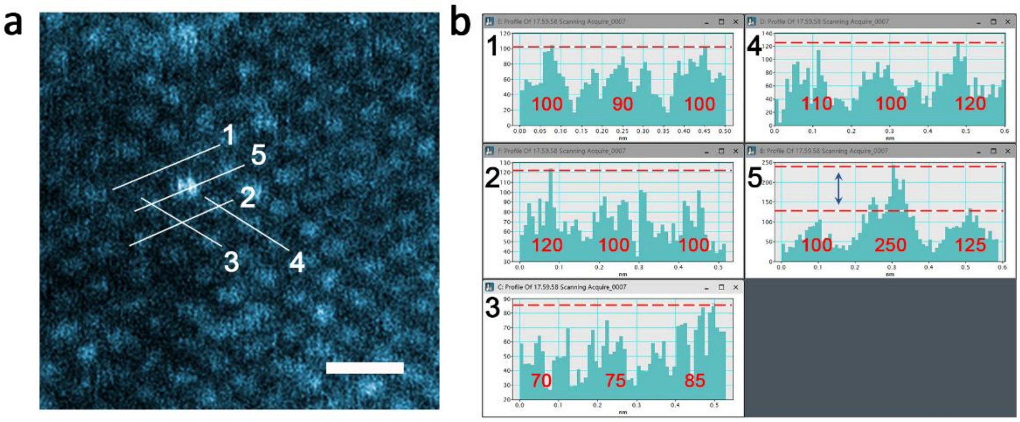

Supplementary Figure 25. Aberration-corrected STEM images. The line profile and corresponding lines of Pb-$CoSe_2$-DETA nanobelts (red characters shows the intensity of each atom). Scale bar, 0.5 nm.

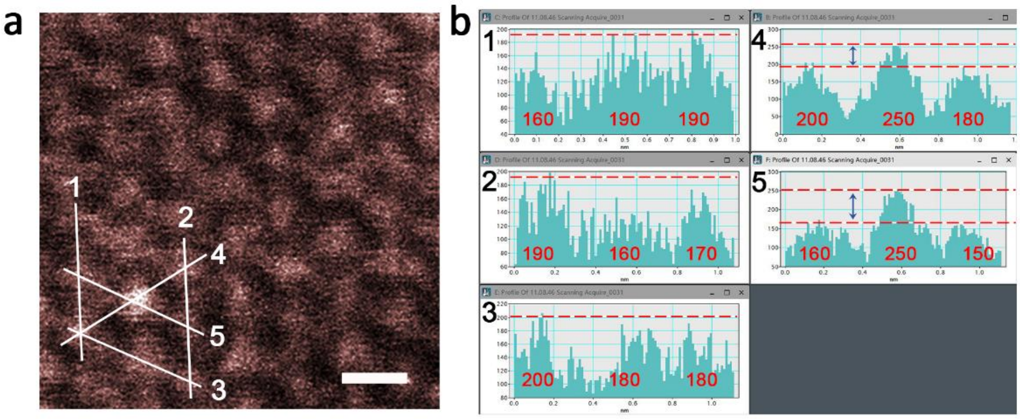

Supplementary Figure 26. Aberration-corrected STEM images. The line profile and corresponding lines of Bi-$CoSe_2$-DETA nanobelts (red characters shows the intensity of each atom). Scale bar, 0.5 nm.

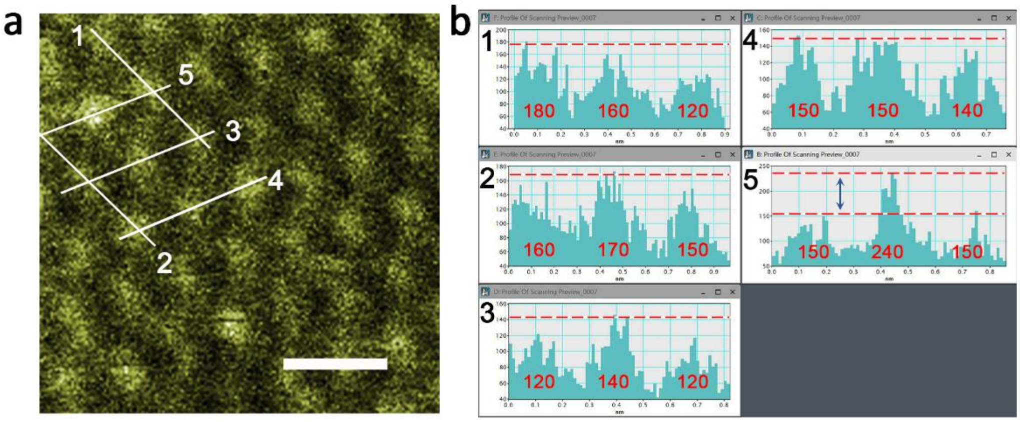

Supplementary Figure 27. Aberration-corrected STEM images. The line profile and corresponding lines of W-$CoSe_2$-DETA nanobelts (red characters shows the intensity of each atom). Scale bar, 0.5 nm.

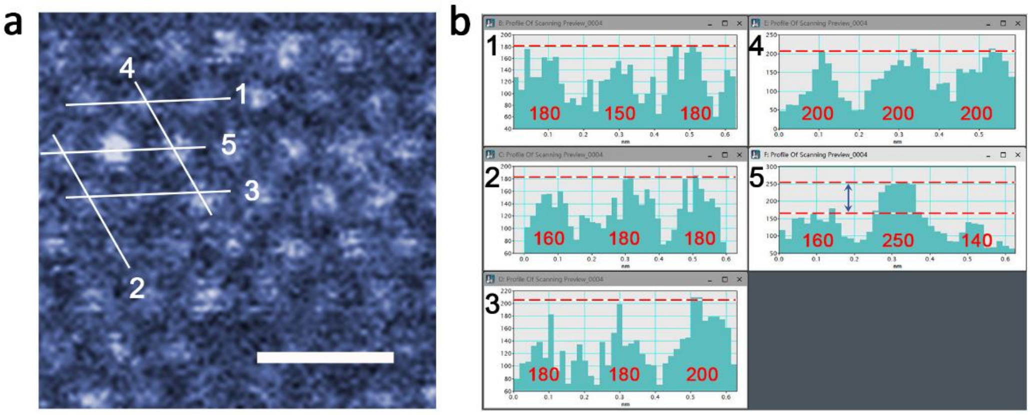

Supplementary Figure 28. Aberration-corrected STEM images. The line profile and corresponding lines of Cd-$CoSe_2$-DETA nanobelts (red characters shows the intensity of each atom). Scale bar, 0.5 nm.

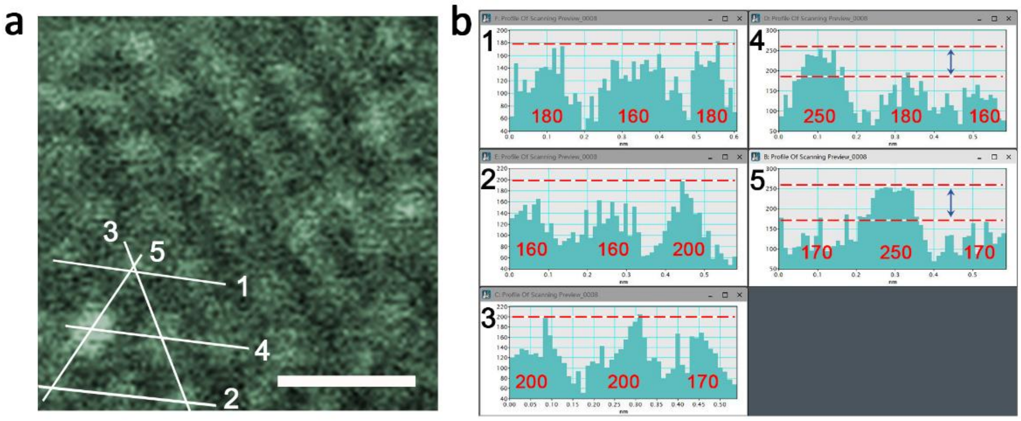

Supplementary Figure 29. Aberration-corrected STEM images. The line profile and corresponding lines of Mo-$CoSe_2$-DETA nanobelts (red characters shows the intensity of each atom). Scale bar, 0.5 nm.

From **Supplementary Figure 25 to 29**, we performed line profiles to show the intensity of bright spots (single atoms) and Se or Co atoms of $CoSe_2$. The intensity of bright spots is clearly much larger than that of Se and Co atoms.

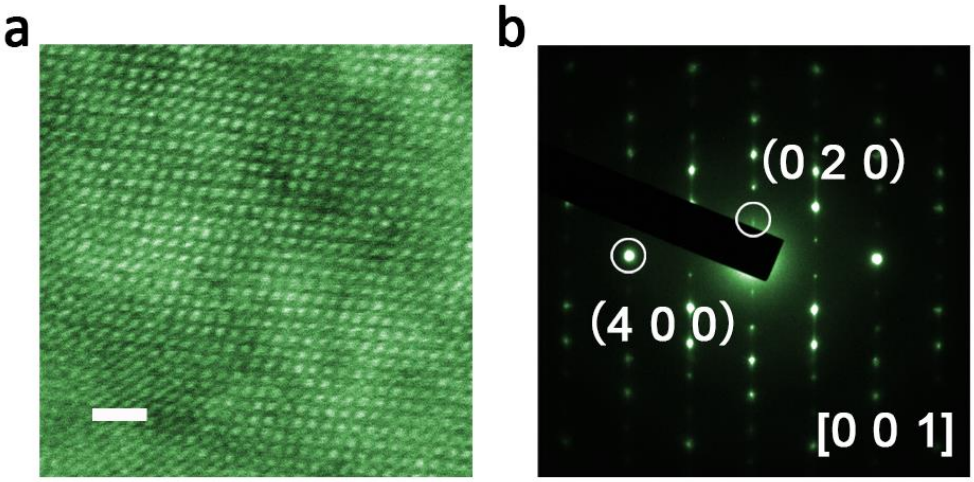

Supplementary Figure 30. STEM images. STEM image (**a**) and SAED pattern (**b**, corresponded to rectangle area in Supplementary Fig. 7) of Zn-$CoSe_2$-DETA nanobelts. Scale bar, 1 nm.

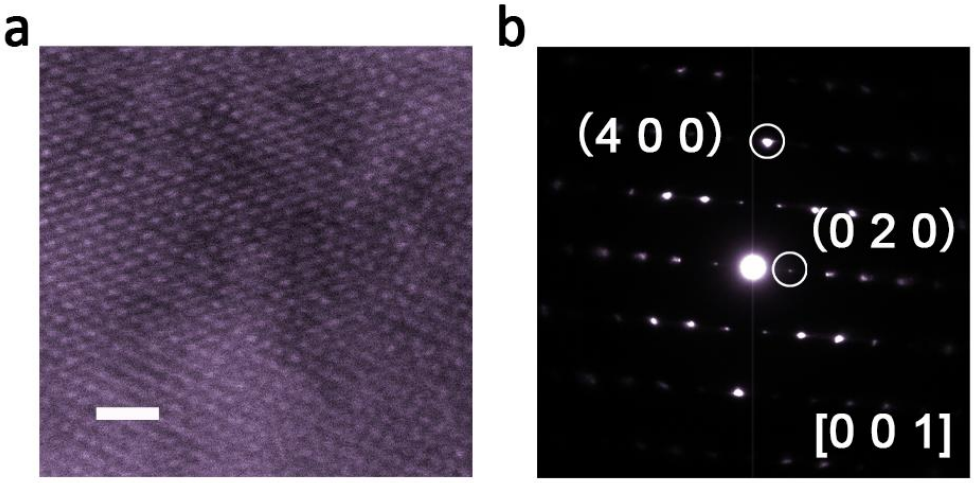

Supplementary Figure 31. STEM images. STEM image (**a**) and SAED pattern (**b**, corresponded to rectangle area in Supplementary Fig. 6) of Ni-$CoSe_2$-DETA nanobelts. Scale bar, 1 nm.

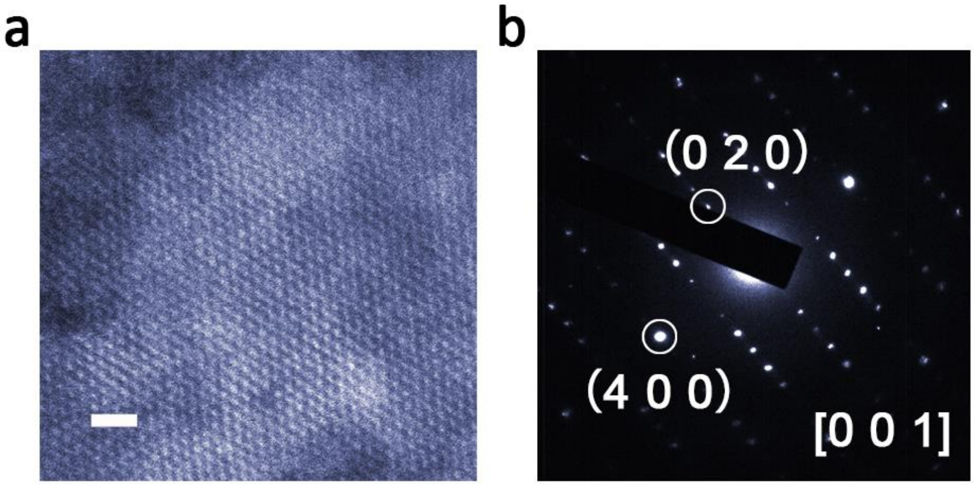

Supplementary Figure 32. STEM images. STEM image (**a**) and SAED pattern (**b**, corresponded to rectangle area in Supplementary Fig. 5) of Fe-$CoSe_2$-DETA nanobelts. Scale bar, 1 nm.

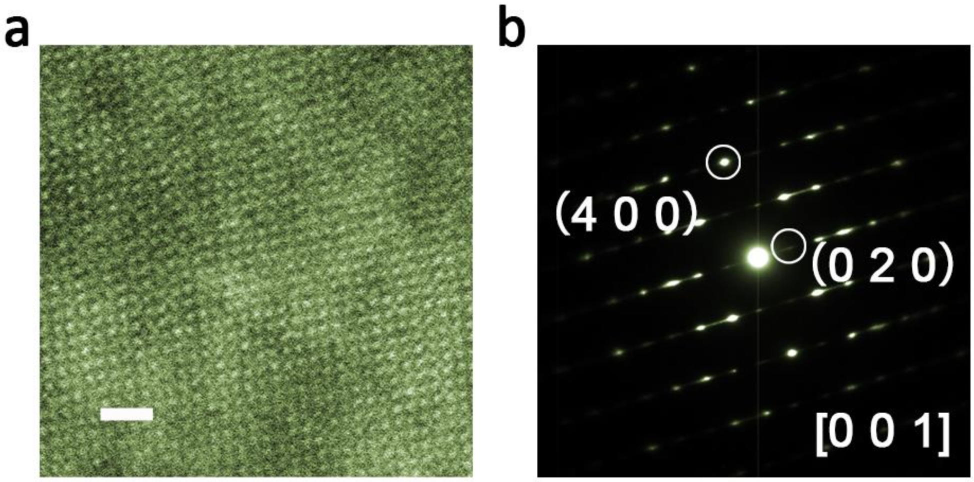

Supplementary Figure 33. STEM images. STEM image (**a**) and SAED pattern (**b**, corresponded to rectangle area in Supplementary Fig. 4) of Mn-$CoSe_2$-DETA nanobelts. Scale bar, 1 nm.

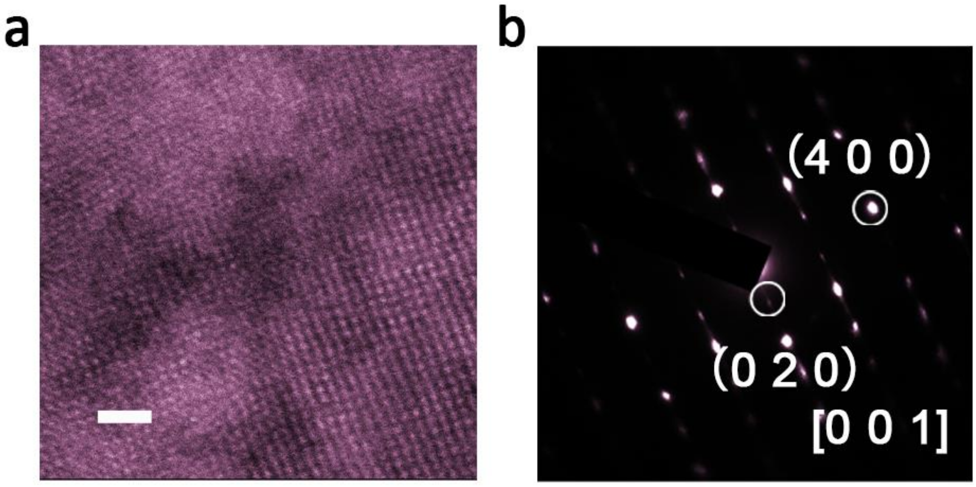

Supplementary Figure 34. STEM images. STEM image (**a**) and SAED pattern (**b**, corresponded to rectangle area in Supplementary Fig. 3) of Cr-$CoSe_2$-DETA nanobelts. Scale bar, 1 nm.

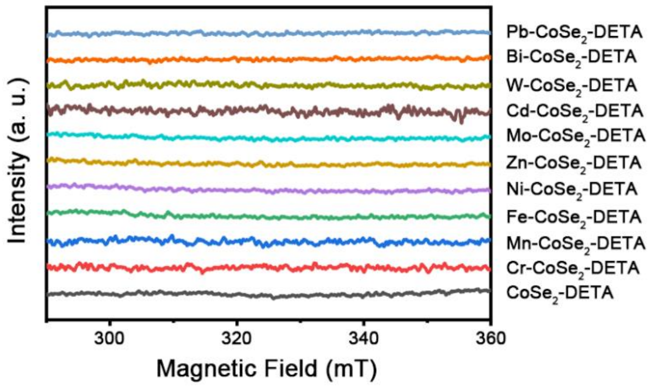

Supplementary Figure 35. EPR spectra. EPR spectra of Pb-, Bi-, W-, Cd-, Mo-, Zn-, Ni-, Fe-, Mn-, Cr-$CoSe_2$-DETA and pure $CoSe_2$-DETA samples (from top to bottom). All samples show no EPR signal, suggesting no vacancies in products.

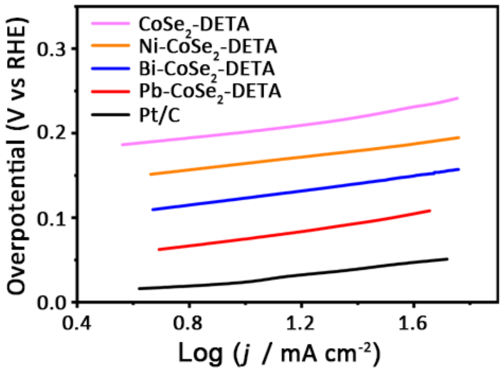

Supplementary Figure 36. Tafel slopes. Tafel slopes for Pb-$CoSe_2$-DETA, Bi-$CoSe_2$-DETA, Ni-$CoSe_2$-DETA, $CoSe_2$-DETA and commercial Pt/C derived from Fig. 4a in the main text. The calculated values are 42, 44, 45, 68 and 31 dec mV^{-1} for Pb-$CoSe_2$-DETA, Bi-$CoSe_2$-DETA, Ni-$CoSe_2$-DETA, $CoSe_2$-DETA and commercial Pt/C, respectively.

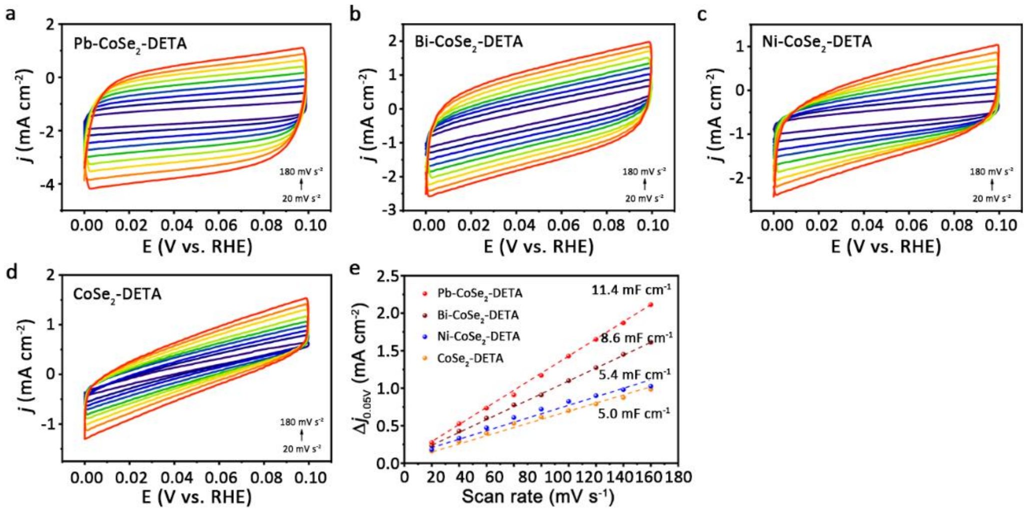

Supplementary Figure 37. C_{dl} tests. CVs of (**a**) Pb-$CoSe_2$-DETA, (**b**) Bi-$CoSe_2$-DETA, (**c**) Ni-$CoSe_2$-DETA and (**d**) $CoSe_2$-DETA in the non-faradic current range at scan rates of 20, 40, 60, 80, 100, 120, 140 and 160 mV s^{-1}. (**e**) Plots showing the C_{dl} for studied catalysts. The C_{dl} values are 11.4, 8.6, 5.4 and 5.0 mF cm^{-1} for Pb-$CoSe_2$-DETA, Bi-$CoSe_2$-DETA, Ni-$CoSe_2$-DETA and $CoSe_2$-DETA, respectively, in order from big go to small, showing the variation of electrochemical active surface areas for studied catalysts.

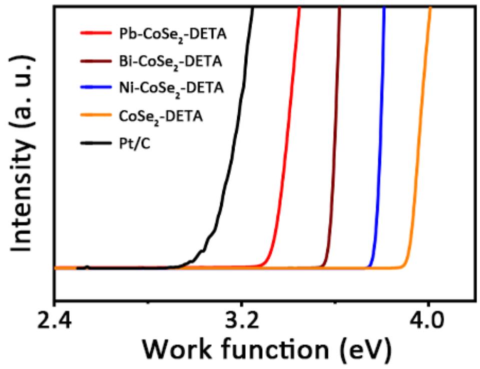

Supplementary Figure 38. UPS tests. UPS spectra of Pb-$CoSe_2$-DETA, Bi-$CoSe_2$-DETA, Ni-$CoSe_2$-DETA, $CoSe_2$-DETA and commercial Pt/C. The calculated values are 3.3, 3.5, 3.8, 3.9 and 2.9 eV for Pb-$CoSe_2$-DETA, Bi-$CoSe_2$-DETA, Ni-$CoSe_2$-DETA, $CoSe_2$-DETA and commercial Pt/C, respectively.

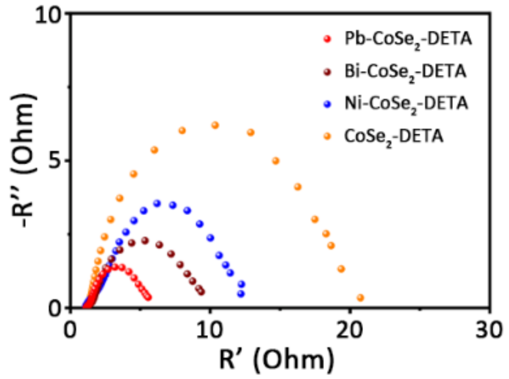

Supplementary Figure 39. EIS nyquist plots. EIS Nyquist plots of Pb-$CoSe_2$-DETA, Bi-$CoSe_2$-DETA, Ni-$CoSe_2$-DETA and $CoSe_2$-DETA.

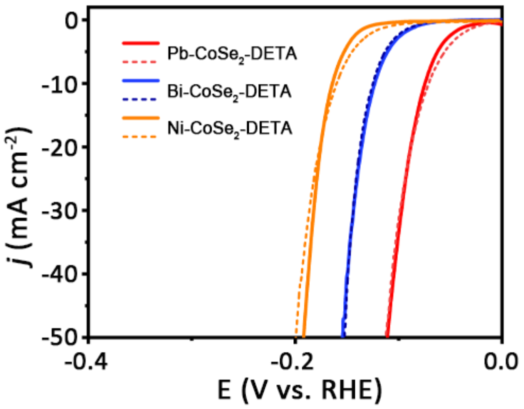

Supplementary Figure 40. Stability tests. Polarization curves of Pb-$CoSe_2$-DETA, Bi-$CoSe_2$-DETA and Ni-$CoSe_2$-DETA before (solid lines) and after (dot lines) 1000 CV cycles. No obvious deactivations were observed for all catalysts.

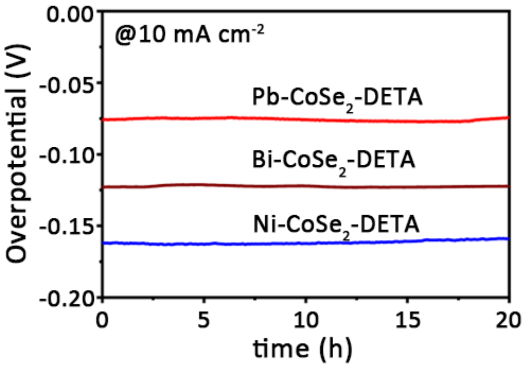

Supplementary Figure 41. Chronoamperometry tests. Long-term stability tests were conducted at constant potentials to achieve 10 mA cm^{-1} for Pb-$CoSe_2$-DETA, Bi-$CoSe_2$-DETA and Ni-$CoSe_2$-DETA, showing no current decay.

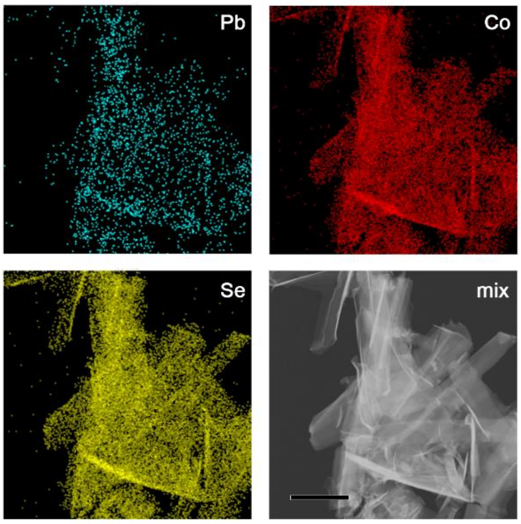

Supplementary Figure 42. HRTEM mapping images. HRTEM mapping images of Pb-$CoSe_2$-DETA after long-term stability test, suggesting no morphological and elemental distribution changes. Scale bar, 200 nm.

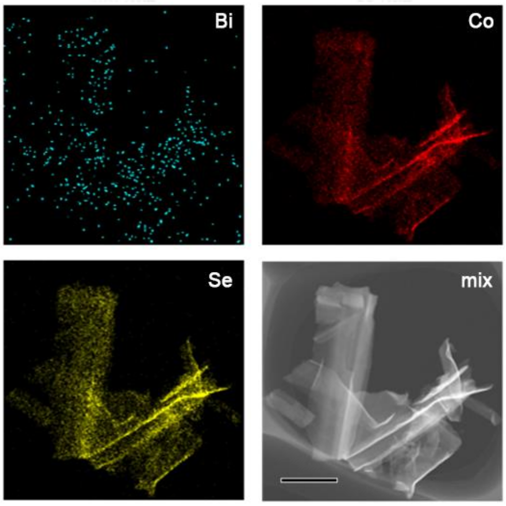

Supplementary Figure 43. HRTEM mapping images. HRTEM mapping images of Bi-$CoSe_2$-DETA after long-term stability test, suggesting no morphological and elemental distribution changes. Scale bar, 100 nm.

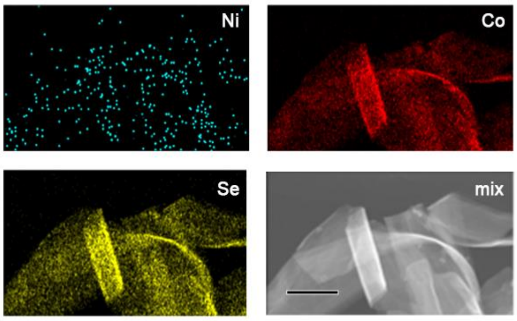

Supplementary Figure 44. HRTEM mapping images. HRTEM mapping images of Ni-$CoSe_2$-DETA after long-term stability test, suggesting no morphological and elemental distribution changes. Scale bar, 100 nm.

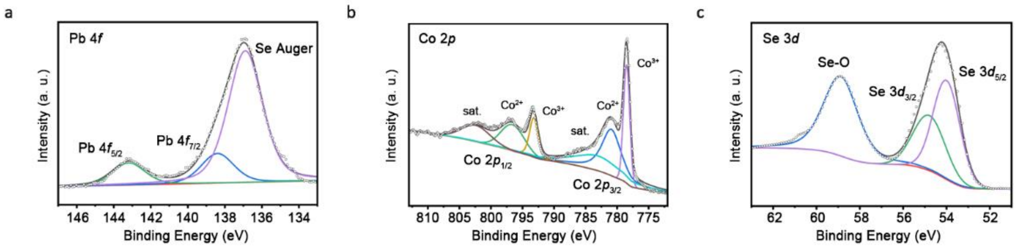

Supplementary Figure 45. XPS spectra. Pb 4*f* spectra (**a**), Co 2*p* spectra (**b**) and Se 3*d* spectra (**c**) of Pb-$CoSe_2$-DETA after long-term stability test.

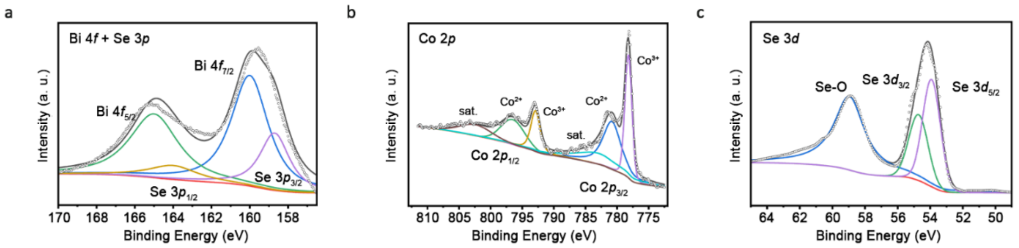

Supplementary Figure 46. XPS spectra. Bi 4*f* spectra (**a**), Co 2*p* spectra (**b**) and Se 3*d* spectra (**c**) of Pb-$CoSe_2$-DETA after long-term stability test.

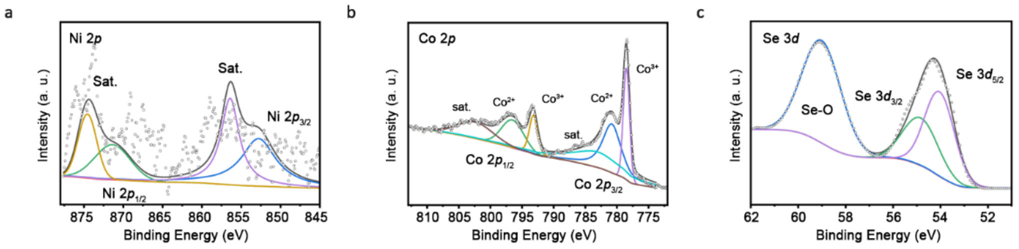

Supplementary Figure 47. XPS spectra. Ni 2*p* spectra (**a**), Co 2*p* spectra (**b**) and Se 3*d* spectra (**c**) of Pb-$CoSe_2$-DETA after long-term stability test.

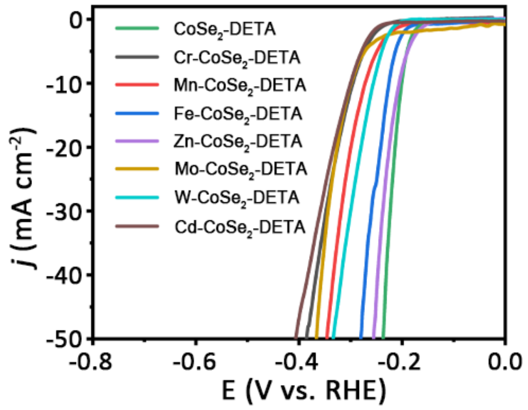

Supplementary Figure 48. HER activity. Polarization curves for Cr-$CoSe_2$-DETA, Mn-$CoSe_2$-DETA, Fe-$CoSe_2$-DETA, Zn-$CoSe_2$-DETA, Mo-$CoSe_2$-DETA, W-$CoSe_2$-DETA, Cd-$CoSe_2$-DETA and $CoSe_2$-DETA.

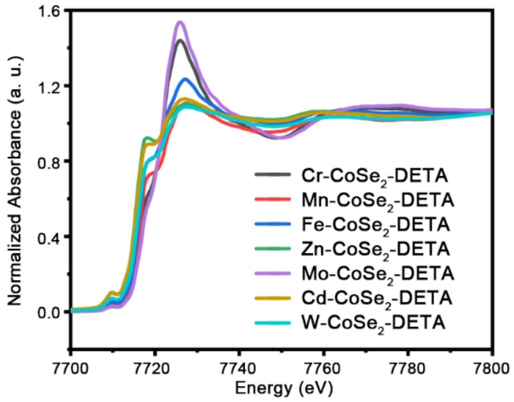

Supplementary Figure 49. Co K-edge EXAFS spectra of Cr-$CoSe_2$-DETA, Mn-$CoSe_2$-DETA, Fe-$CoSe_2$-DETA, Zn-$CoSe_2$-DETA, Mo-$CoSe_2$-DETA, Cd-$CoSe_2$-DETA and W-$CoSe_2$-DETA samples.

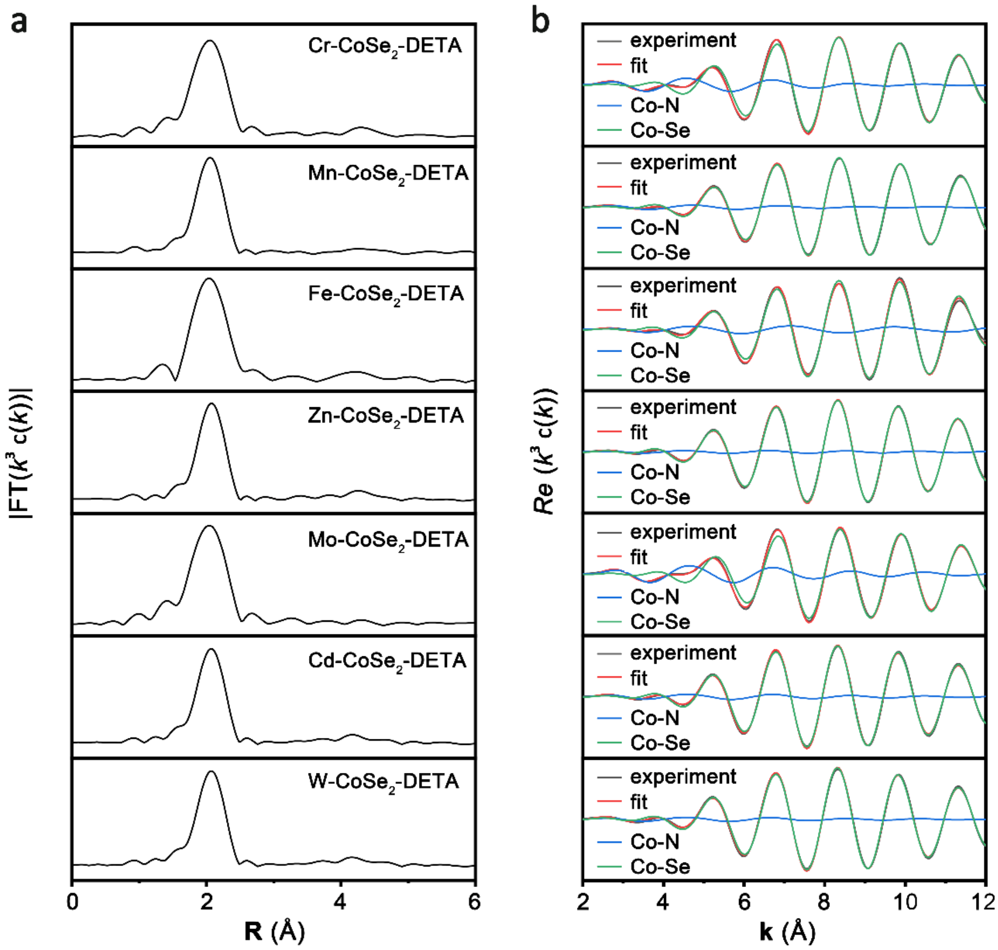

Supplementary Figure 50. EXAFS analysis. R-space curve-fitting of EXAFS spectra (**a**) and corresponding Re($k^3\chi(k)$) oscillations (**b**) of Co-N and Co-Se (b) of Cr-$CoSe_2$-DETA, Mn-$CoSe_2$-DETA, Fe-$CoSe_2$-DETA, Zn-$CoSe_2$-DETA, Mo-$CoSe_2$-DETA, Cd-$CoSe_2$-DETA and W-$CoSe_2$-DETA samples.

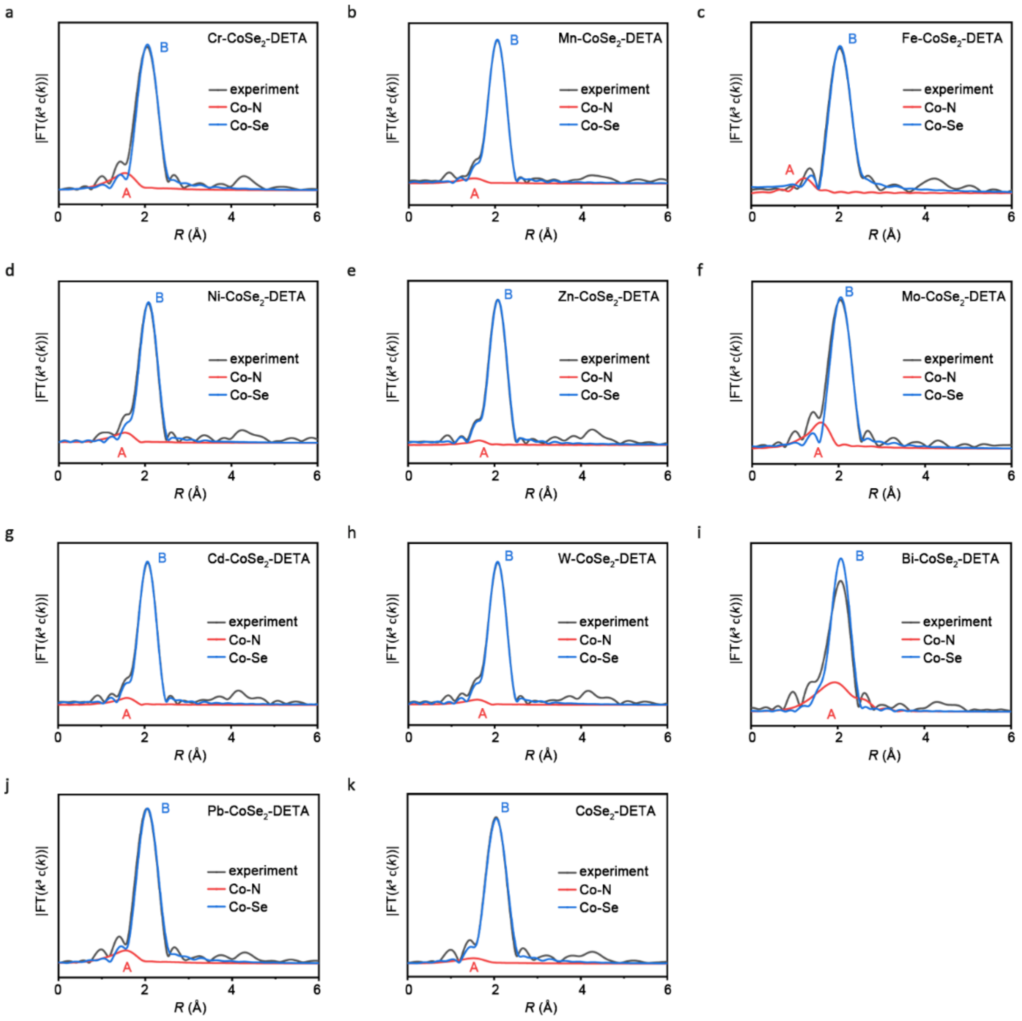

Supplementary Figure 51. EXAFS analysis. R-space curve-fitting of EXAFS spectra considering Co-N and Co-Se of Cr-$CoSe_2$-DETA, Mn-$CoSe_2$-DETA, Fe-$CoSe_2$-DETA, Ni-$CoSe_2$-DETA, Zn-$CoSe_2$-DETA, Mo-$CoSe_2$-DETA, Cd-$CoSe_2$-DETA, W-$CoSe_2$-DETA, Bi-$CoSe_2$-DETA, Pb-$CoSe_2$-DETA and $CoSe_2$-DETA samples. Peak A is assigned to Co-N coordinates and peak B is assigned to Co-Se coordinates.

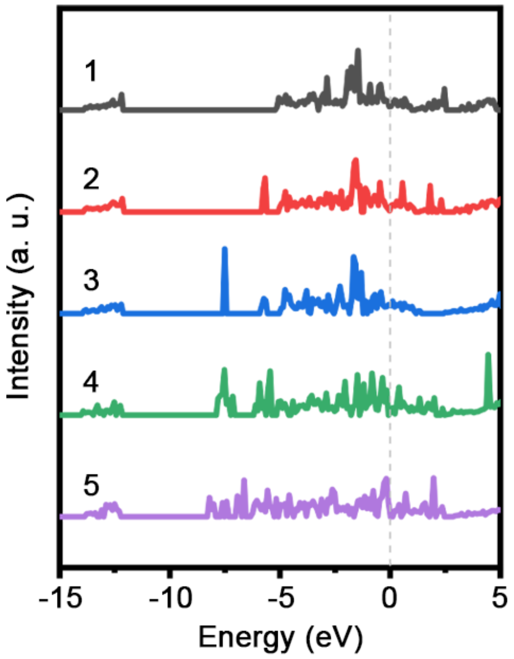

Supplementary Figure 52. Calculated projected density of states of studies DFT models. DFT model, 1, 2, 3, 4, 5, $-NH_2$ absorbed on the surface of $CoSe_2$ lattice with Co-N to Co-Se ratio of 0.1, 0.2, 0.35, 0.5 and 0.6, respectively.

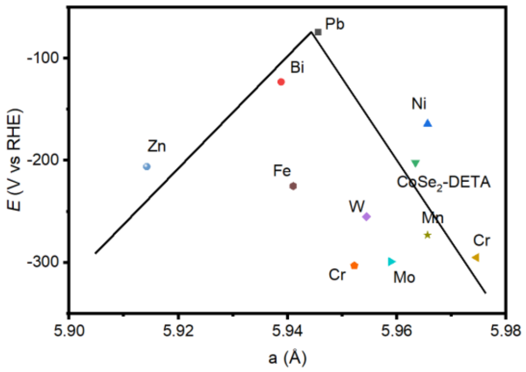

Supplementary Figure 53. Lattice parameter-HER activity dependency. The relationship between overpotentials at 10 mA cm^{-2} and calculated a value. A volcano line is shown for guidance.

As to the cubic $CoSe_2$ (belonging to isometric system), its lattice parameter a=b=c. We used the strongest diffraction peaks located around 33.4 degree that assigned to (2 1 0) crystal face (Supplementary Figure 1) to calculate their lattice parameters, according to following equation:

$$d = \frac{a}{\sqrt{h^2 + k^2 + l^2}}$$

in which h=2, k=1, l=0.

Based on the calculations, we obtained the calculated lattice parameter (a) for each studied sample (Supplementary Table 5). We plotted the overpotentials at 10 mA cm^{-2} as function of a (Supplementary Figure 53). While several samples don't locate within the volcano line range (such

as Fe-$CoSe_2$-DETA and Cr-$CoSe_2$-DETA), most samples show a good relationship between HER activity and lattice parameter. This proves the lattice parameter might be another HER activity descriptor like previous report (Ref. 49 in the main text). Such relationship only suggests another tool, which won't be discussed in depth here.

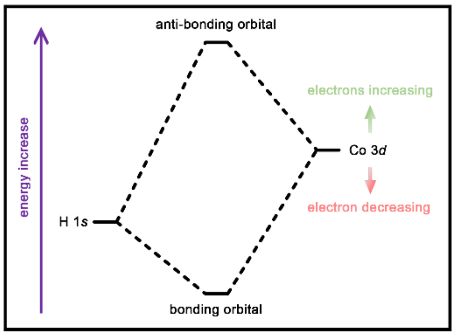

Supplementary Figure 54. Schematic diagram of H-Co bonding.

Supplementary Table 1. DFT-calculated defect formation energies of M-$CoSe_2$-DETA.

DFT system	ΔE_f (eV)	Lattice distortion (%)
pure $CoSe_2$	0.00	0.0
Pb-$CoSe_2$	2.19	1.9
W-$CoSe_2$	0.87	0.53
Cd-$CoSe_2$	1.14	1.4
Mo-$CoSe_2$	0.29	0.50
Ni-$CoSe_2$	-0.07	0.040
Cr-$CoSe_2$	0.12	0.21

ΔE_f represents the calculated formation energies with the value of pure $CoSe_2$ to be 0 eV. Lattice distortion is the ratio of volume change before and after doping. Dopant concentration is 3.125 at%.

Supplementary Table 2. EXAFS data fitting results of single-atom dopants (M) for M-$CoSe_2$-DETA.

Sample	Shell	N	R_j (Å)	σ^2 (10^{-3} Å^2)	E_0 (eV)	R factor
1	M-Co	3.3	3.32	0.0090	9.25	0.003
2	M-N	5.5	2.00	0.0033	-1.1	0.012
	M-Co	4.8	2.94	0.0073	-4.8	0.012
3	M-N	5.0	2.21	0.0052	-3.8	0.020
	M-Co	3.1	3.32	0.0056	-0.3	0.020
4	M-N	4.3	2.03	0.0075	0.9	0.002
	M-Co	5.5	2.99	0.0089	7.5	0.002
5	M-N	2.1	2.06	0.0051	1.90	0.015
	M-Se	3.1	2.43	0.0050	2.76	0.015
6	M-N	5.0	1.75	0.0030	-4.8	0.009

Sample, 1, Pb-$CoSe_2$-DETA 2, Cr-$CoSe_2$-DETA, 3, Mn-$CoSe_2$-DETA, 4, Fe-$CoSe_2$-DETA, 5, Zn-$CoSe_2$-DETA, 6, Mo-$CoSe_2$-DETA; R, distance between center and backscatter atoms; σ^2, the Debye-Waller factor value; E_0, inner potential correction to account for the difference in inner potential between the sample and the reference compound.

Supplementary Table 3. EXAFS data fitting results of Co for M-$CoSe_2$-DETA.

Sample	Shell	N	R_j (Å)	σ^2 (10^{-3} Å^2)	E_0 (eV)	R factor
1	Co-N	2.2	2.02	0.0104	-2.49	0.0033
	Co-Se	3.6	2.39	0.0067	-2.49	0.0033
2	Co-N	0.7	2.01	0.0048	0.75	0.0128
	Co-Se	4.3	2.40	0.0076	0.75	0.0128
3	Co-N	0.6	2.04	0.0016	-2.91	0.0047
	Co-Se	3.6	2.38	0.0055	-2.91	0.0047
4	Co-N	1.1	2.00	0.0099	-1.30	0.0073
	Co-Se	4.3	2.39	0.0066	-1.30	0.0073
5	Co-N	0.3	2.03	0.0045	-1.60	0.0025
	Co-Se	5.3	2.40	0.0074	-1.60	0.0025
6	Co-N	1.9	2.03	0.0097	1.11	0.0026
	Co-Se	3.3	2.41	0.0064	1.11	0.0026
7	Co-N	0.6	2.03	0.0070	-1.11	0.0022
	Co-Se	5.4	2.41	0.0087	-1.11	0.0022
8	Co-N	0.5	2.04	0.0085	0.28	0.0033
	Co-Se	5.4	2.41	0.0085	0.28	0.0033
9	Co-N	1.5	2.01	0.0048	0.75	0.0128
	Co-Se	3.3	2.40	0.0072	0.75	0.0128
10	Co-N	1.6	2.04	0.0130	-1.82	0.0056
	Co-Se	4.4	2.39	0.074	-1.82	0.0056
11	Co-N	0.9	2.03	0.170	-2.7	0.0053
	Co-Se	5.1	2.39	0.082	-2.7	0.0053

Sample, 1, Cr-$CoSe_2$-DETA, 2, Mn-$CoSe_2$-DETA, 3, Fe-$CoSe_2$-DETA, 4, Ni-$CoSe_2$-DETA, 5, Zn-$CoSe_2$-DETA, 6, Mo-$CoSe_2$-DETA, 7, Cd-$CoSe_2$-DETA, 8, W-$CoSe_2$-DETA, 9, Bi-$CoSe_2$-DETA, 10, Pb-$CoSe_2$-DETA, 11, $CoSe_2$-DETA; R, distance between center and backscatter atoms; σ^2, the Debye-Waller factor value; E_0, inner potential correction to account for the difference in inner potential between the sample and the reference compound.

Supplementary Table 4. DFT calculated ΔE_{H^*} and ΔG_{H^*} values of H* adsorbed on Co site and Co Bader charges of studied catalysts surfaces.

DFT Model	ΔE_{H^*} (eV)	ΔG_{H^*} (eV)	Bader charge (eV)
1	0.58	0.82	8.533
2	0.17	0.41	8.330
3	-0.12	0.12	8.320
4	0.21	0.45	8.279
5	0.48	0.72	8.239

DFT model, 1, 2, 3, 4, 5, $-NH_2$ absorbed on the surface of $CoSe_2$ lattice with Co-N to Co-Se ratio of 0.1, 0.2, 0.35, 0.5 and 0.6, respectively.

Supplementary Table 5. Summary of the lattice parameter of each sample.

	sample	2 theta degree of (2 1 0) peak	a (Å)
1	Cr-$CoSe_2$-DETA	33.50	5.97
2	Mn-$CoSe_2$-DETA	33.56	5.96
3	Fe-$CoSe_2$-DETA	33.70	5.94
4	Ni-$CoSe_2$-DETA	33.56	5.96
5	Zn-$CoSe_2$-DETA	33.86	5.91
6	Mo-$CoSe_2$-DETA	33.60	5.96
7	Cd-$CoSe_2$-DETA	33.64	5.95
8	W-$CoSe_2$-DETA	33.62	5.95
9	Bi-$CoSe_2$-DETA	33.72	5.94
10	Pb-$CoSe_2$-DETA	33.68	5.94
11	$CoSe_2$-DETA	33.58	5.96
